# A Comparative Study on Luminescence Properties of Y_2_O_3_: Pr^3+^ Nanocrystals Prepared by Different Synthesis Methods

**DOI:** 10.3390/nano10081574

**Published:** 2020-08-11

**Authors:** Andrea Diego-Rucabado, Marina T. Candela, Fernando Aguado, Jesús González, Fernando Rodríguez, Rafael Valiente, Rosa Martín-Rodríguez, Israel Cano

**Affiliations:** 1 Applied Physics Department, University of Cantabria, 39005 Santander, Spain; andrea.diego@unican.es (A.D.-R.); marinateresa.candela@unican.es (M.T.C.); rafael.valiente@unican.es (R.V.); 2 CITIMAC Department, University of Cantabria, 39005 Santander, Spain; fernando.aguado@unican.es (F.A.); jesusantonio.gonzalez@unican.es (J.G.); fernando.rodriguez@unican.es (F.R.); 3 Nanomedicine Group, IDIVAL, 39011 Santander, Spain; 4 QUIPRE Department, University of Cantabria, 39005 Santander, Spain

**Keywords:** emission lifetime, luminescence, nanocrystal, praseodymium, yttria, Y_2_O_3_: Pr^3+^

## Abstract

Pr^3+^-doped Y_2_O_3_ nanocrystals (NCs) have been obtained via five wet-chemistry synthesis methods which were optimized in order to achieve superior optical properties. To this end, a systematic study on the influence of different reaction parameters was performed for each procedure. Specifically, precursor concentration, reaction temperature, calcination temperature, and time, among others, were analyzed. The synthesized Y_2_O_3_: Pr^3+^ NCs were characterized by differential scanning calorimetry (DSC), thermogravimetric analysis (TGA), powder X-ray diffraction (PXRD), transmission electron microscopy (TEM), and reflectance and Raman spectroscopy. In addition, the optical properties of such NCs were investigated by excitation, emission, and luminescence decay measurements. Concretely, emission from the ^1^D_2_ level was detected in all samples, while emission from ^3^P_J_ was absent. Finally, the effect of the synthesis methods and the reaction conditions on the luminescence decay has been discussed, and a comparative study of the different methods using the fluorescence lifetime of so-obtained Y_2_O_3_: Pr^3+^ NCs as a figure of merit has been carried out.

## 1. Introduction

Crystalline nanomaterials have gained increasing popularity in recent years due to their noteworthy applications in important research fields like biomedicine and catalysis, and their straightforward preparation in comparison to the production of single crystals. Specifically, rare-earth doped nanocrystals (RE NCs) have drawn remarkable attention in the past few decades due to their singular properties such as high photostability, narrow emission lines, long fluorescence lifetimes, and easy functionalization [1,2]. As a result, a wide variety of applications has arisen in different fields, including medicine [3] and biology [4], solar energy [5], data storage [6], and optoelectronics [7]. Amid all the aforementioned fields, optics could be considered the most popular one due to the noteworthy number of applications, like fiber lasers and amplifiers [8], cathode ray tubes [9], plasma display panels [10], field emission displays [11], or fluorescence lamps [12] to name a few. Among all the available host materials used to prepare RE NCs, yttrium sesquioxide (Y_2_O_3_), also known as yttria, has proven to be a very suitable compound owing to its high thermal and chemical stability as well as its good optical and mechanical properties. Besides, its broad transparency range and low phonon energy favor low non-radiative relaxation rates for RE doping systems [13]. Specifically, Y_2_O_3_: Pr^3+^ NCs exhibit efficient red luminescence from the ^1^D_2_ level, while the resulting emission from ^3^P_0_ is completely absent [14,15] which is a common characteristic of all sesquioxides with cubic structure [15,16] and results in a potential application as a red phosphor [17].

A large number of studies have been described on luminescent RE-doped yttrium oxides, mainly based on Eu^3+^ [18,19,20,21,22], Er^3+^ [23,24,25], Yb^3+^ [26,27,28], and Tb^3+^ [29,30,31]. However, the number of publications on Pr^3+^ is scarce despite its potential application in telecommunications, since the 1.3 μm emission can be used as an amplifier in the second telecom window. In this context, the generation of Y_2_O_3_: Pr^3+^ NCs has been accomplished by physical methods such as laser heated pedestal growth (LHPG) technique [32], flame-fusion or Verneuil method [33], or the use of a xenon arc image furnace with argon atmosphere to grow Pr^3+^-doped yttria crystals [34]. Alternatively, chemical synthetic routes like thermal decomposition [15], solvent evaporation [35], co-precipitation [14], or sol–gel method [17] have also been employed. Interestingly, these studies show remarkable differences in the luminescent properties of analyzed Y_2_O_3_: Pr^3+^ NCs. Structural factors such as nanocrystal size, dopant concentration, crystalline single phase, or absence of defects and impurities on the top of the NCs surface affect their optical properties, particularly the emission intensity and its associated lifetime. The NC size can be tuned and the presence of surface contamination can be reduced by modifying the synthesis procedure [36,37]. Thus, the influence of the synthesis method on luminescence exhibited by the Y_2_O_3_: Pr^3+^ NCs deserves a systematic analysis and can be extrapolated to many other host lattices.

In view of the lack of studies on the influence of the synthesis procedure on the luminescence properties of RE-doped NCs, in this work we analyze in detail Y_2_O_3_: 0.1%Pr^3+^ NCs prepared through five different wet-chemistry methods. The luminescence properties of the so-obtained NCs are compared to provide an insight into the factors that lead to different spectroscopic behaviors. It has been previously reported that 0.1% Pr^3+^ concentration provides the best optical properties, namely the longest luminescence decay rates, pointing out the absence of RE clustering, and therefore concentration quenching of luminescence [22]. Although following the described procedures is possible to reduce the final NC size, we are interested in NCs large enough to ‘survive’ to certain thermal treatments when they are embedded in different host matrixes. At the same time, these NCs must be small enough to reduce light scattering processes. In this context, our aim is to prepare NCs with sizes in the range of 20–60 nm.

The synthesized Y_2_O_3_: Pr^3+^ NCs have been characterized by several experimental techniques, such as differential scanning calorimetry, thermogravimetric analysis, powder X-ray diffraction, and transmission electron microscopy. Additionally, a spectroscopic characterization has been carried out through reflectance and Raman spectroscopy. Finally, the excitation and emission spectra and fluorescence lifetime of all NCs have been measured and investigated.

## 2. Experimental

### 2.1. General Procedures

Ethanol (>99.8%, Sigma-Aldrich, St. Louis, United States) was purchased as HPLC grade and deionized water was used. Urea (99.5%, Merck, Kenilworth, United States), KNO_3_ (>98%, Panreac, Barcelona, Spain), NaNO_3_ (>99%, Panreac, Barcelona, Spain), citric acid (>99%, Alfa Aesar, Ward Hill, United States), ethylenediaminetetraacetic acid (EDTA, >99.995%, Sigma-Aldrich), polyethylene glycol (Merck), and ethylene glycol (Scharlab, Barcelona, Spain) were purchased from commercial sources. The same precursors were used for all synthesis procedures. Both yttrium and praseodymium nitrates were purchased from Strem Chemicals (Newburyport, United States) as the hexahydrate form with 99.9% purity. For the sake of simplicity, they will be referred as Y(NO_3_)_3_ and Pr(NO_3_)_3_, respectively. All chemicals were used without any further purification. All manipulations were performed in air atmosphere.

### 2.2. Instrumentation

#### 2.2.1. Powder X-Ray Diffraction (PXRD)

The crystal structure of all synthesized samples was checked by PXRD, whereas the analysis of the peak widths provided estimates of the average particle size. PXRD measurements were performed in a Bruker D8 Advanced diffractometer equipped with a Cu tube (wavelength: <Kα_1,2_> = 1.5418 Å) and a fast LYNXEYE 1D-detector. The synthesized NCs were typically measured in the 10°–120° range (2θ) for both phase identification and structural refinements. PXRD patterns were analyzed by the Rietveld method using the TOPAS software package [38]. In addition, double-Voigt approach [39] was used for estimating average crystallite size and effective strain of all the samples. Instrumental contribution was taken into account by the fundamental parameters approach (FPA) [40].

#### 2.2.2. Transmission Electron Microscopy (TEM)

Morphology and size distribution were analyzed through TEM images obtained with a JEOL JEM 1011 equipped with a high-resolution CCD camera (Gatan, Pleasanton, United States). NCs were dispersed in ethanol and a small drop was deposited on the copper grid.

#### 2.2.3. Thermogravimetric Analysis (TGA) and Differential Scanning Calorimetry (DSC)

TGA and DSC measurements were performed on a Setaram Setsys evolution TGA-DTA/DSC model coupled with a mass spectrometer Pfeiffer OmniStar. The measurements were performed heating the samples from room temperature (RT) up to 900 °C at a heating rate of 10 °C/min in argon atmosphere.

#### 2.2.4. Confocal Micro-Raman Spectroscopy

Unpolarized confocal micro-Raman measurements were performed by means of a triple monochromator (Horiba-Jobin-Yvon, Model T64000), in subtractive-mode backscattering configuration, equipped with a liquid-N_2_-cooled CCD detector. The 488 nm line of an Ar^+^−Kr^+^ laser was focused on the sample with a 20× objective, and the laser power was kept below 4 mW in order to avoid laser-heating effects. The laser spot was 2 μm in diameter, and the spectral resolution was better than 0.6 cm^−1^ for all spectra.

#### 2.2.5. Reflectance Spectroscopy

The RT absorption spectra of synthesized samples were obtained in the 200–1800 nm spectral range with a Cary 6000i equipped with an integrating sphere (DRA 1800), coated with polytetrafluoroethylene (PTFE), which exhibits NIR superior performance compared to traditional coatings, maintaining UV–vis capabilities. Absorption measurements of the NCs were made relative to a baseline using PTFE as a standard reference material.

#### 2.2.6. Luminescence Spectroscopy

Emission and excitation spectra as well as lifetime measurements were obtained for all samples with a FLSP920 spectrofluorometer from Edinburgh Instrument equipped with double monochromators in emission and excitation, different excitation sources, a 450 W Xe lamp, and a pulsed lamp of 60 W. The light was detected with an electrically cooled photomultiplier tube R928P (Hamamatsu, Shizuoka, Japan).

### 2.3. Synthesis of Y_2_O_3_: Pr^3+^ Nanocrystals

Pr^3+^-doped Y_2_O_3_ NCs were prepared by five different methods: combustion [41], molten-salt [42], sol–gel Pechini [43], homogeneous precipitation [44], and solvothermal [45]. Different parameters were optimized to achieve the best performance in terms of optical quality, which is related to Pr^3+^ distribution and luminescent quencher concentration. The following procedures describe the synthesis conditions for 0.1 mol% Pr^3+^ concentration. The Pr^3+^ content for all samples refers to nominal concentrations.

#### 2.3.1. Combustion Method

Y(NO_3_)_3_ (2.61 mmol), Pr(NO_3_)_3_ (0.0026 mmol) and urea (6.82 mmol) were dissolved in deionized water (3 mL) in a beaker, which was covered with a watch glass. The solution was heated up at 500 °C for 10 min. Then, the so-obtained Y_2_O_3_: Pr^3+^ NCs were calcined at 900 °C for 4 h using a ramp of 5 °C/min.

#### 2.3.2. Molten Salt Method

Y(NO_3_)_3_ (7.84 mmol), Pr(NO_3_)_3_ (0.0078 mmol), NaNO_3_, and KNO_3_ (235.2 mmol each, 30 eq. regarding total amount of RE) were ground in an agate mortar for 15 min. The well-mixed powder was then heated up at 500 °C in a ceramic crucible for 3 h with a ramp of 5 °C/min. After cooling down to RT, the so-obtained solid was washed with deionized water followed by centrifugation (5–10 times) until no crystallization of salts was observed in the supernatant (5–10 × 100 mL). Afterwards, the purified Y_2_O_3_: Pr^3+^ NCs were dried overnight (o.n.) at 100 °C.

#### 2.3.3. Sol–Gel Pechini Method

Y(NO_3_)_3_ (20 mmol) and Pr(NO_3_)_3_ (0.02 mmol) were dissolved in deionized water (200 mL) under stirring. Then, citric acid or EDTA (2 eq. regarding amount of lanthanides) was added over this solution. The resulting mixture was heated up at 90 °C for 15 min. Afterwards, polyethylene glycol (2.01 mmol) was added to the solution, which was stirred at 90 °C for 15 min. The obtained sol was kept at 90 °C for 24 h without stirring, leading to the formation of a gel. Finally, the gel was fired at the chosen temperature (800 or 900 °C) for the required time (16 or 24 h).

#### 2.3.4. Homogeneous Precipitation Method

Y(NO_3_)_3_ (1.43 mmol) and Pr(NO_3_)_3_ (0.0014 mmol) were dissolved in deionized water (3 mL). This solution was added to a well-stirred solution of a large excess of urea in deionized water. Next, the mixture was heated up at 85 °C for the chosen time. Afterwards, the reaction mixture was cooled down to RT and the solid was washed with deionized water and centrifuged several times (3 × 100 mL). The solid was then suspended in EtOH (50 mL) to avoid the aggregation of preformed NCs and dried o.n. at 60 °C. Finally, the solid was calcined at 800 °C for 3 h with a ramp of 5 °C/min.

#### 2.3.5. Solvothermal Method

Y(NO_3_)_3_ (5.04 mmol) and Pr(NO_3_)_3_ (0.005 mmol) were dissolved in a mixture of ethanol or ethylene glycol and deionized water (19:1). The solution was heated up while stirring in order to dissolve the lanthanide salts. Then, the solution was introduced into a Teflon-lined stainless-steel autoclave, sealed and heated up at the chosen temperature (180 or 220 °C) for 24 h. Then, the reaction mixture was naturally cooled down to RT and the so-obtained solid was washed and centrifuged with EtOH:H_2_O (1:1) (1 × 100 mL) and deionized water (3 × 100 mL). Finally, the solid was dried o.n. at 70 °C and calcined at the chosen temperature (800, 900, and 1000 °C) for the required time (4 or 8 h) with a ramp of 5 °C/min.

## 3. Results and Discussion

The aim of this work is to evaluate the effect of the NC size and purity on the Pr^3+^ optical properties by analyzing in detail NCs prepared through five different Y_2_O_3_: Pr^3+^ synthesis procedures. Firstly, the crystalline structure and particle size is studied by PXRD and Raman spectroscopy, and the presence of Pr^3+^ is confirmed by reflectance spectroscopy. The influence of the synthesis method itself as well as diverse parameters, such as precursors ratio or calcination temperature, on the crystal size, size distribution, and particle aggregation has been investigated by TEM. Secondly, a thorough analysis of the Y_2_O_3_: Pr^3+^ NCs optical properties has been performed by means of emission and excitation spectra. Clearly, luminescence from Pr^3+^ ions in both C_2_ and S_6_ crystallographic sites is observed for all samples. Finally, the Pr^3+^ emission lifetime as a function of the NCs synthesis method is used as a figure of merit, as well as the dependence of the emission lifetime on Pr^3+^ concentration. The luminescence decay time is an appropriate parameter to calibrate the homogeneous distribution of Pr^3+^ and the presence of impurity traps acting as luminescence quenchers. Since all NCs have been synthesized with the same precursors, the only effect is due to the synthesis method and conditions. 

### 3.1. Synthesis and Structural Characterization of Y_2_O_3_: Pr^3+^ Nanocrystals

**Figure 1:**
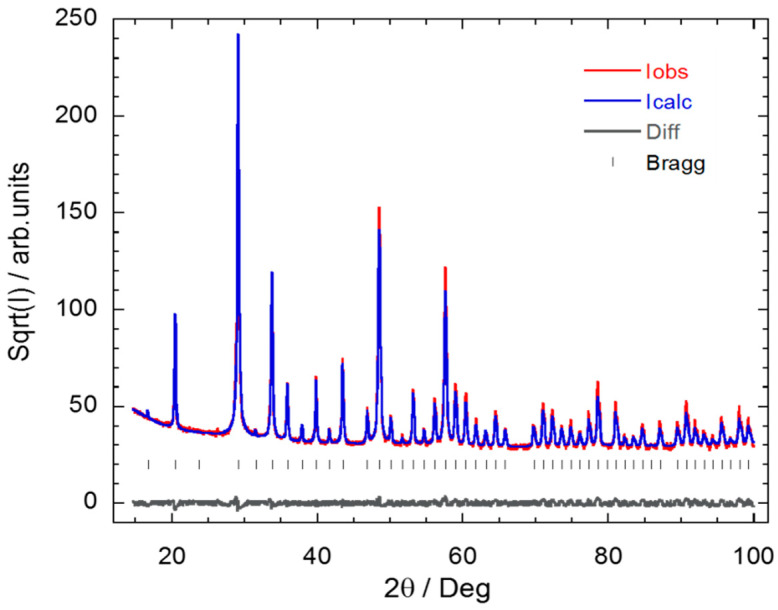
Rietveld refinement results on the Y_2_O_3_: Pr^3+^ NCs obtained by solvothermal method at 220 °C followed by calcination at 1000 °C for 4 h. Only a single cubic phase (S.G. *Ia*$\bar{3}$) was used for the refinement, providing a good fitting (*R_B_* = 3.6). No impurity phases could be detected within the experimental uncertainty. Vertical lines correspond to Bragg reflections.

All the so-obtained Y_2_O_3_: Pr^3+^ NCs were initially studied by PXRD, and reflectance and Raman spectroscopy. The PXRD pattern analyses indicate that the five synthesis methods provide the same crystalline structure (Figure 1 and Figure S1, ESI). Indeed, all the Y_2_O_3_: Pr^3+^ NCs samples show a pure cubic phase (space group *Ia*$\bar{3}$) in which two different crystallographic sites, C_2_ (75%) and S_6_ (25%), can be occupied by Pr^3+^ ions [46]. In both sites, Pr^3+^ is present in a six-fold coordination surrounded by oxygen ions [15]. As the structure is body-centered, the unit cell contains twice the primitive cell. The Y^3+^ ions occupy two types of Wyckoff positions, 8b and 24d, whereas all O ions occupy the 48e position.

From the crystal space group, 120 vibrational modes are possible. The irreducible representations for the optic and acoustic vibrational modes are [47]

Γ_op_= 4 A_g_ + 4 E_g_ +14 T_g_ + 5 A_2u_ + 5 E_u_ + 16 T_u_ (1)

Γ_ac_ = T_u_ (2)

**Figure 2:**
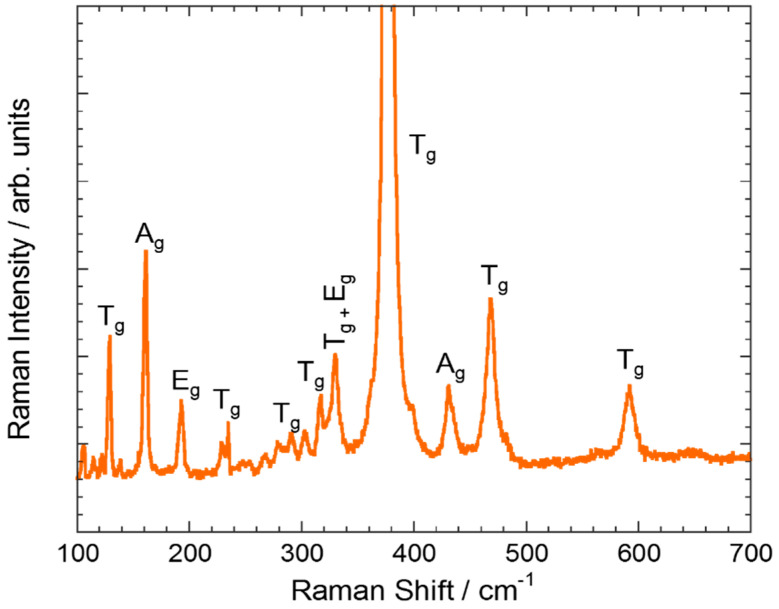
Raman spectrum of Y_2_O_3_: Pr^3+^ NCs obtained by solvothermal method at 220 °C followed by calcination at 1000 °C for 4 h. Only the most intense peaks were assigned.

Among these 120 modes, there are 51 grouped into 17 infrared active modes of T_u_ symmetry and 54 into 22 Raman active modes (4A_g_ + 4E_g_ + 14T_g_). Consequently, up to 22 lines would be expected in the Raman spectra. However, a smaller number of lines was experimentally observed, probably due to the superposition of different types of transitions. All the samples exhibit similar Raman spectra (Figure 2 and Figure S2, ESI). The Raman peaks observed in the 0–600 cm^−1^ range were assigned according to refs. [47,48], being the most intense one located at around 376.4 cm^−1^. This band corresponds to a T_g_ vibration type. On the other hand, the peaks observed in the region of the external lattice vibrations—i.e., below 200 cm^−1^—are associated to pure Y^3+^ vibrations [47].

Table 1 shows the Raman frequencies, peak width (FWHM) and their symmetry assignment. The data were obtained by fitting to Lorentzian functions. No relationship between FWHM and nanocrystal size or synthesis method was found within the experimental resolution (0.6 cm^−1^). According to dispersion curves [49], a displacement of the most prominent band (Figure S3, ESI), placed at *ca*. 376.4 cm^−1^, towards lower frequencies is expected upon decreasing the nanocrystal size. However, this peak does not present any displacement independently of the synthesis method or nanocrystal size, and thus there is no confinement effect due to the size.

**Figure 3:**
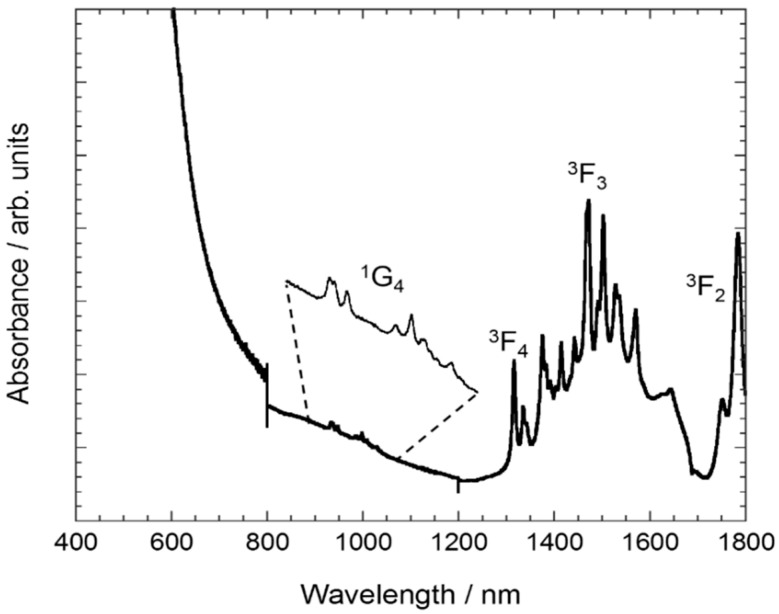
Absorption spectrum of Y_2_O_3_: Pr^3+^ NCs obtained by sol–gel Pechini method using citric acid as chelating agent and followed by thermal treatment at 900 °C for 16 h.

In addition, the reflectance spectrum of all samples is dominated by a broad band centered below 400 nm (Figure 3 and Figure S4, ESI), which is associated with interconfigurational 4*f*^2^ → 4*f*^1^5*d*^1^ transitions of Pr^3+^ ions hosted in the C-Y_2_O_3_ lattice. Low-intensity and sharp peaks are assigned to *f-f* intraconfigurational transitions. The transition from the ground state to the ^1^G_4_ excited state is weakly observed at *ca*. 1000 nm. The absorption peaks detected from 1300 to 1800 nm correspond to transitions from the ^3^H_4_ ground state to the ^3^F_J_ multiplets [15]. There is a jump in absorbance intensity at 800 nm due to the change of detector and diffraction grating that is not corrected by the baseline correction.

Thus, PXRD as well as Raman and reflectance spectroscopy confirm that Y_2_O_3_ NCs doped with Pr^3+^ obtained via the five synthesis methods of choice present the same crystalline structure and vibrational peaks. It is worth mentioning that these synthesis methods produce NCs with sizes in the 20–60 nm range. This size range can minimize the surface effects due to Pr^3+^ ions located at the NCs surface. In the following sub-sections, an exhaustive analysis of particle size, size distribution, and aggregation of NCs is performed by means of TEM images analysis. This is very relevant to understand the optical properties of the as-prepared nanoparticles.

#### 3.1.1. Combustion Method

**Figure 4:**
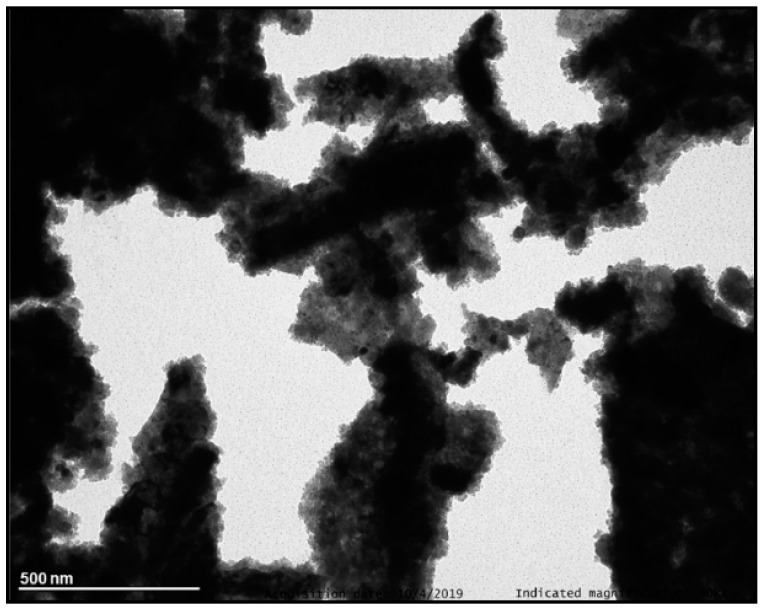
TEM image of Y_2_O_3_: Pr^3+^ NCs prepared by combustion method after calcination at 900 °C for 4 h.

The Y_2_O_3_: Pr^3+^ NCs prepared by combustion method were calcined at 900 °C to improve the crystallinity. TEM images (Figure 4) revealed the presence of highly agglomerated NCs with a grain size in the 10–40 nm range. This NC size is in good agreement with the one estimated by PXRD (35.7(5) nm), thus indicating single domain NCs. Additionally, TGA (Figure S5a, ESI) showed a small weight loss of *ca*. 1.5% from RT to 400 °C related to the presence of reagent traces.

#### 3.1.2. Molten Salt Method

**Figure 5:**
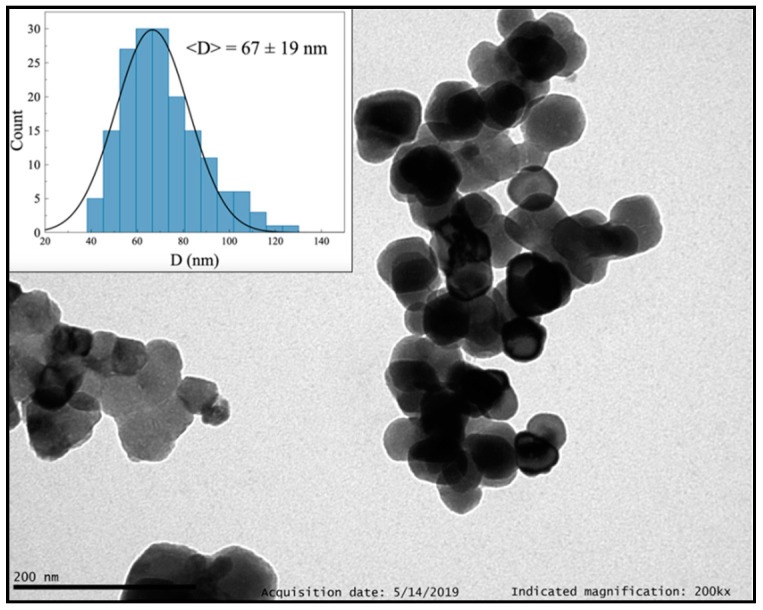
TEM image of Y_2_O_3_: Pr^3+^ NCs prepared by molten salt method after 10 washing cycles.

Y_2_O_3_: Pr^3+^ NCs obtained via molten salt method look polyhedral and well dispersed, with an average size of 67(19) nm (Figure 5 and Figure S6, ESI). PXRD analysis (Figure S1b, ESI) showed a good crystallinity grade and an average grain size of 77.7(4) nm, which was compatible with TEM observations. TG + DSC measurements (Figure S5b, ESI) displayed several weight losses from RT to 800 °C that decreased with the increase in the number of washing cycles, and thus could be a sign of the presence of remaining NaNO_3_ and KNO_3_ salts.

#### 3.1.3. Sol–Gel Pechini Method

**Figure 6:**
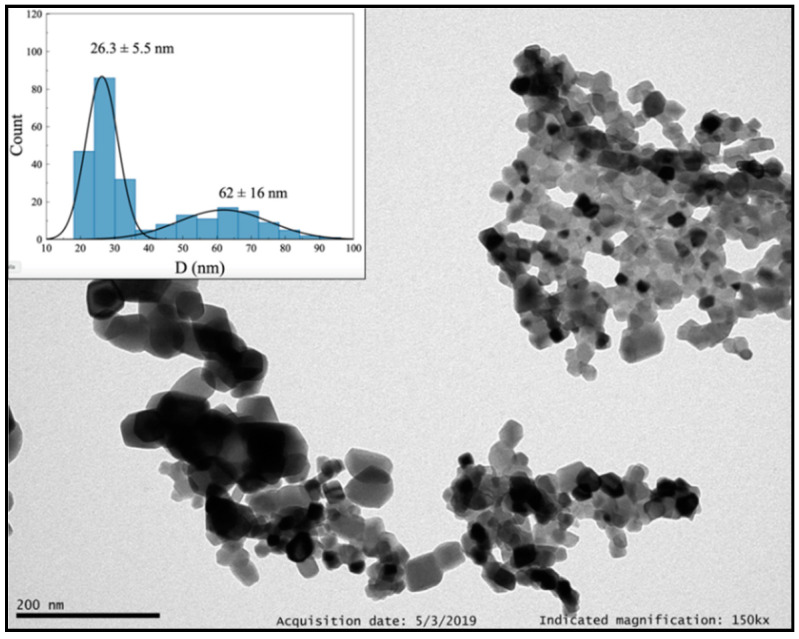
TEM image of Y_2_O_3_: Pr^3+^ NCs obtained by sol–gel Pechini method using citric acid as chelating agent and followed by thermal treatment at 900 °C for 16 h.

Different parameters were modified to optimize the synthesis of Y_2_O_3_: Pr^3+^ NCs by sol–gel Pechini method, specifically the type of hydroxyl carboxylic acid or chelating agent, as well as temperature and calcination time (Table 2). Firstly, citric acid was selected as chelating agent (Entry 1). The formed gel was calcined at 800 °C for 16 h, leading to the formation of NCs surrounded by a presumably organic layer (Figure S7, ESI). No changes were observed by TEM when the calcination time was increased up to 24 h (Entry 2 and Figure S8, ESI). However, such a layer was removed by increasing the calcination temperature up to 900 °C (Entry 3), as confirmed by TEM and TG + DSC analyses (Figure 6 and Figures S9 and S5c, ESI). Under these conditions, the synthesized NCs are polyhedral crystallites (Figure S1c, ESI) and show two populations of different size, 26(6) and 62(16) nm. Indeed, the average grain size determined by PXRD was 51(9) nm. Finally, EDTA was used instead of citric acid (Entry 4). The obtained NCs are more homogenous in size, 40(12) nm, but exhibit agglomeration (Figure S10, ESI).

#### 3.1.4. Homogeneous Precipitation Method

In general, the homogeneous precipitation method led to the generation of small crystalline NCs with grain size in the 20–30 nm range forming spherical nanoparticles with an excellent dispersion. Firstly, spheres of 260(26) nm (and a few of 106(14) nm) containing NCs with 20.2(2) nm in size (Figure S11, ESI) were observed using the following reaction conditions: 360 mL of solvent, 0.485 mol of urea and 2 h as reaction time (Table 3, entry 1). Subsequent synthesis reactions were carried out by modifying the water volume, which was increased up to 720 mL and reduced to 200 mL (Entries 2 and 3). In both cases, the nanospheres presented appropriate dispersion and well-defined spherical morphology, while the NC size distribution hardly changed and was 21.8(3) and 25.2(4) nm, respectively. In addition, the sphere size showed no appreciable differences, with an average size of 233(41) nm when H_2_O volume was doubled and 229(20) nm when it was reduced to 200 mL (Figures S12 and S13, ESI). Consequently, it seems that the water volume does not affect the size of NCs and nanospheres nor the dispersion. Similarly, the reaction time had no influence on these properties, since no changes were observed for reaction times of 45, 60, 90, 120, and 180 min.

**Figure 7:**
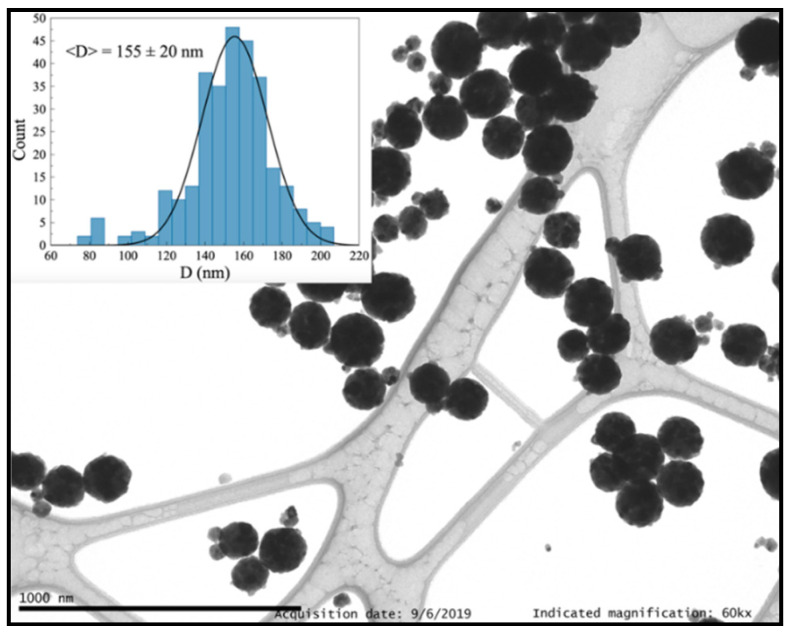
Y_2_O_3_: Pr^3+^ nanoparticles synthesized via homogeneous precipitation using 200 mL of deionized water and 0.832 mmol of urea.

Interestingly, a change in the urea content produced remarkable differences, not only in size but also in nanoparticle dispersion. A clear tendency was confirmed by PXRD analysis and TEM images, in which an increase in the amount of urea led to a reduction of nanoparticle size. Indeed, NCs synthesized using 0.166 mol of urea (Entry 4) showed a grain size of 26.9(3) nm and average sphere size of 338(35) nm (Figure S14, ESI). In contrast, an average NC size of 22.2(2) nm and an average spherical nanoparticle size of 75(34) nm were observed with 1.415 mol of urea (Entry 5 and Figure S15, ESI). In addition, these spherical nanoparticles exhibit a broad size distribution. An intermediate urea concentration (0.832 mol) provided NCs of 21.1(2) nm and spheres with 219(24) nm in size (Entry 6 and Figure S16, ESI). On the other hand, the dispersion displayed an inverse trend; that is, samples prepared with low urea concentrations presented an excellent dispersion of nanospheres (Figure S14, ESI), while high urea amounts led to slightly agglomerated nanoparticles (Figure S15, ESI). In this sense, we were interested in finding the conditions that promote not only an appropriate size and good dispersion of the formed spheres but also a narrow particle size distribution, which will lead to a better understanding of the NCs optical performance. In this context, Y_2_O_3_: Pr^3+^ NCs with a grain size of 22.4(3) nm, an average sphere size of 155(20) nm and excellent dispersion (Figure 7 and Figures S17 and S18, ESI) were obtained by the use of 200 mL of deionized H_2_O and 0.832 mol of urea (Entry 7). Moreover, no organic or precursor traces were found by TG + DSC analysis (Figure S5d, ESI).

#### 3.1.5. Solvothermal Method

Finally, we synthesized Y_2_O_3_: Pr^3+^ NCs through the solvothermal method. The synthesis process required heating the reaction mixture at 180 °C in a Teflon-lined stainless-steel autoclave. Then, the resulting sample was calcined at 800 °C for 4 h (Table 4, entry 1). These conditions led to the formation of polyhedral NCs that exhibit some aggregation and 31(7) nm in size (Figure S19, ESI), while the grain size determined by PXRD was 21.1(1) nm. Additionally, an organic layer was observed. This was confirmed by a TGA in which several weight losses were identified below 500 °C. Consequently, the calcination temperature was increased up to 900 °C in order to suppress this layer (Entry 2). The resulting NCs showed a polyhedral morphology and an increase in size up to 42(11) nm (Figure S20, ESI), which was consistent with PXRD analysis (31.1(3) nm). Besides, the organic layer was successfully removed. The effect of additional parameters was also studied. The concentration of RE precursors was doubled (Entry 3), which resulted in a considerable increase in the NC aggregation, although the polyhedral morphology was not altered (Figure S21, ESI). PXRD analysis showed a grain size of 43.1(3) nm, which was in good agreement with that observed by TEM (40(10) nm), indicating NCs with single domain structure. The effect of the employed alcohol was also examined, and thus EG was replaced by EtOH (Entry 4). TEM images showed NCs with an average size of 28(10) nm and remarkable aggregation. Furthermore, the presence of the above-mentioned organic layer was also detected (Figure S22, ESI). On the other hand, the calcination time was increased up to 8 h (Entry 5), which led to a strong aggregation (Figure S23, ESI). To sum up, high concentrations of precursors, the use of other alcohols and longer calcination times produce an increase in NC aggregation.

**Figure 8:**
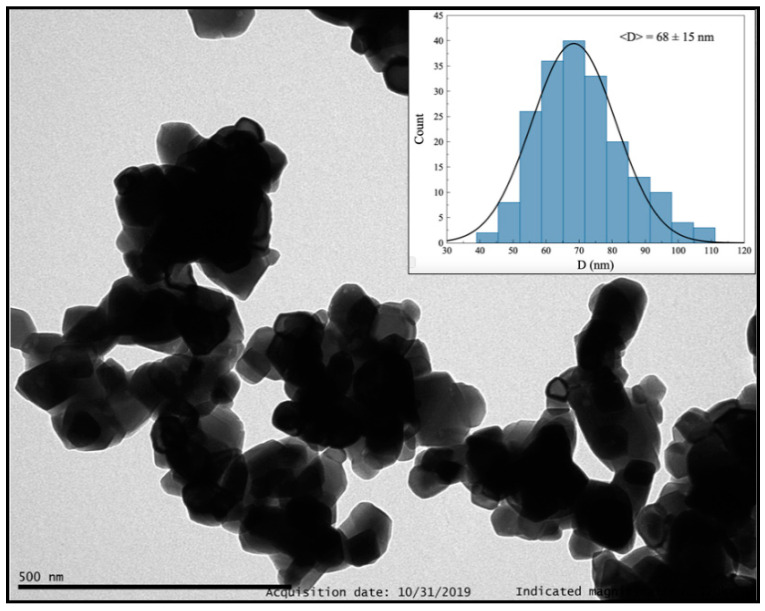
TEM image of Y_2_O_3_: Pr^3+^ NCs obtained by solvothermal method at 220 °C followed by calcination at 1000 °C for 4 h.

Finally, the influence of the temperature was also analyzed. An increase in both reaction and calcination temperatures generally leads to bigger NCs. Specifically, an increase in the autoclave temperature from 180 °C to 220 °C (Entry 6) provided NCs of 47(12) nm in size, while no organic layer was observed (Figure S24, ESI). Next, maintaining the reaction temperature at 220 °C, the calcination temperature was increased up to 1000 °C (Entry 7). These conditions promoted a great increase in the average NC size up to 68(15) nm (Figure 8 and Figure S25, ESI), while that determined by PXRD was 52.8(5) nm. Additionally, TEM images (Figure 8 and Figure S25, ESI) and TG + DSC analysis (Figure S5e, ESI) confirmed that no organic contamination was present.

### 3.2. Optical Properties of Synthesized Y_2_O_3_: Pr^3+^ Nanocrystals

**Figure 9:**
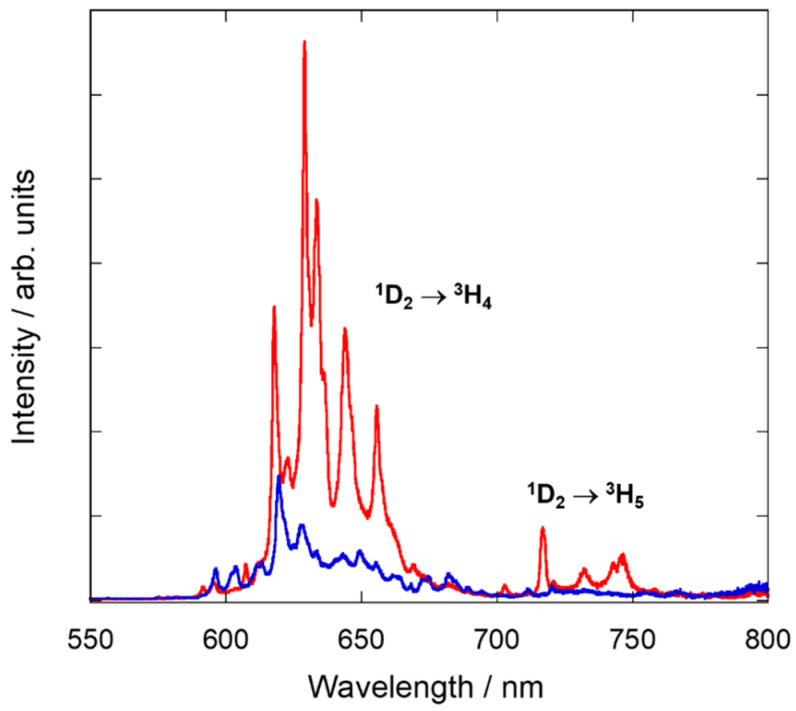
Emission spectra from C_2_ (red, λ_ex_ = 292 nm) and S_6_ (blue, λ_ex_ = 330 nm) sites at RT of Y_2_O_3_: Pr^3+^ NCs prepared by solvothermal method at 220 °C followed by calcination at 1000 °C for 4 h.

**Figure 10:**
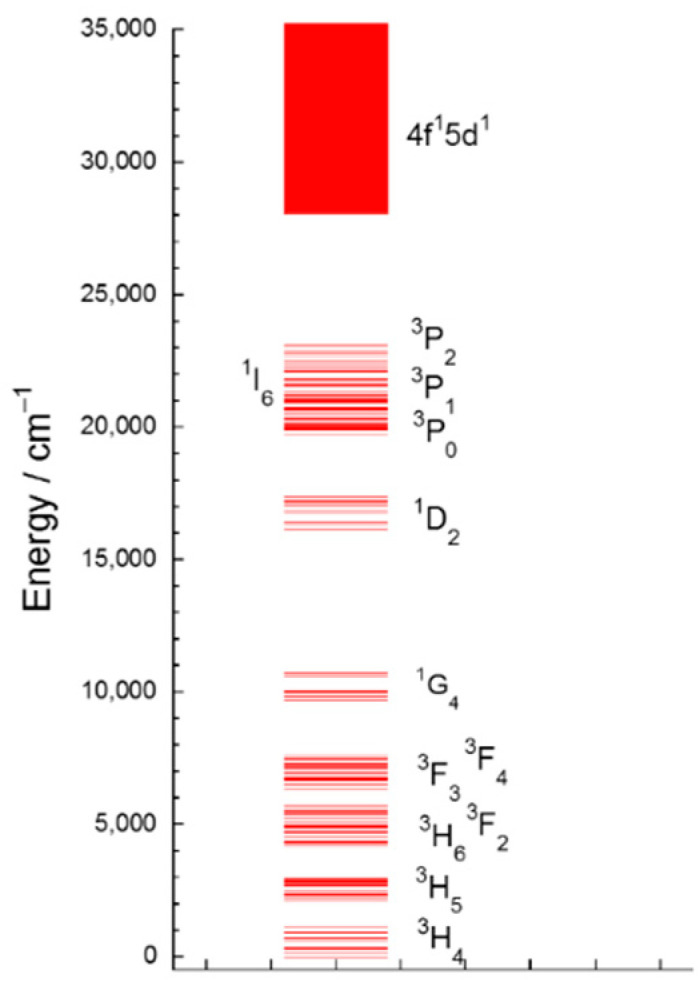
Energy level diagram below 35,000 cm^−1^ of Pr^3+^-doped Y_2_O_3_ obtained from the absorption and emission spectra.

To analyze the optical properties of the NCs prepared following the five aforementioned synthesis procedures, emission and excitation spectra were recorded for selected samples. RT emission spectra were studied upon direct excitation of Pr^3+^ in the UV (at 292 and 330 nm) or 490 nm into the ^3^P_J_ multiplet (for both sites) (Figure 9 and Figures S26 and S27, ESI). The two UV excitation wavelengths allowed us to record two different emission spectra, which we associated to Pr^3+^ placed at C_2_ and S_6_ sites, respectively. The intensity of the Pr^3+^ emission spectrum at the C_2_ site is about an order of magnitude more intense than the one corresponding to the S_6_, in agreement with the 3:1 ratio of the two sites and the absence of inversion center for the C_2_ site. All samples were analyzed under the same conditions in terms of experimental setup and geometry. The NCs prepared by five different synthesis procedures present similar emission spectra, with only slight differences regarding luminescence intensity. All emission spectra exhibit peaks between 600 and 670 nm which are characteristic of emission from ^1^D_2_ to ^3^H_4_ level, while peaks located in the range of 700–750 nm are assigned to the transition ^1^D_2_ → ^3^H_5_ (Figure 10).

The differences observed in emission intensities for the NCs can be related to features such as Pr^3+^ distribution, size, surface effects, and crystallinity. As an example, it is worth mentioning that the luminescence intensity shown by NCs prepared through Pechini method is about an order of magnitude more intense than that displayed by Y_2_O_3_: Pr^3+^ NCs obtained via molten salt synthesis (Figure S26 and S27, ESI). Nevertheless, the luminescence intensity is a difficult magnitude to compare among samples and therefore the luminescence lifetime (τ) was chosen as a more accurate parameter to perform a proper comparison of the optical properties, since it does not depend on the geometry, configuration of the experimental setup, and grain size.

**Figure 11:**
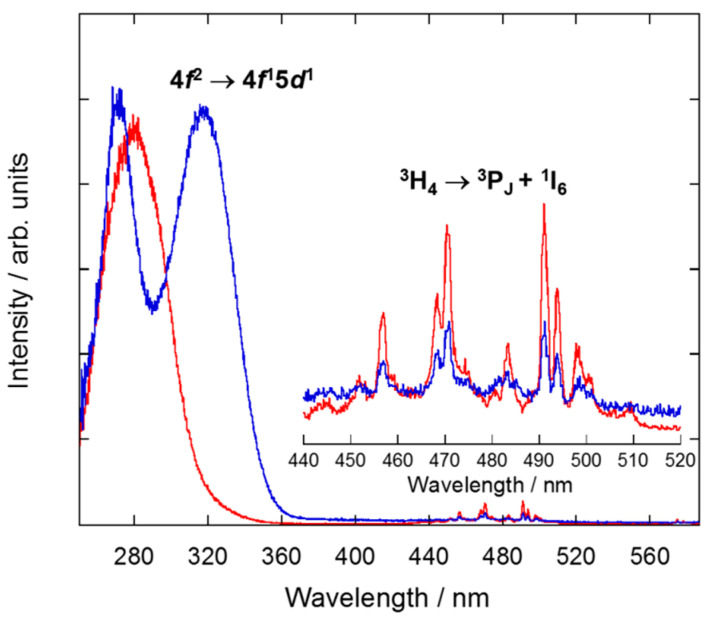
Excitation spectra at C_2_ and S_6_ (red, λ_em_ = 717 nm; blue, λ_em_ = 603 nm, respectively, see Figure 9) sites of Y_2_O_3_: Pr^3+^ NCs prepared by solvothermal method at 220 °C followed by calcination at 1000 °C for 4 h. The inset shows a zoom of the intraconfigurational *f-f* transitions of Pr^3+^ ions.

The excitation spectra were also studied monitoring the emission from the ^1^D_2_ multiplet to the ^3^H_4_ ground state (λ_em_ = 717 and 603 nm for C_2_ and S_6_, respectively). All synthesized Pr^3+^-doped Y_2_O_3_ NCs showed similar excitation spectra (Figure 11 and Figures S28 and S29, ESI), dominated by broad bands in the UV region resulting from the interconfigurational transition 4*f*^2^ → 4*f*^1^5*d*^1^ of Pr^3+^, since yttria presents no absorption in this below-gap spectral range [15]. According to Aumüller et al. [14], this transition occurs at higher energies at C_2_ sites than at S_6_ sites for sesquioxides. Thus, the broad excitation band centred at 280 nm is assigned to Pr^3+^ ions at C_2_ whereas the peak at 317 nm is assigned to Pr^3+^ ions at S_6_ site. Additionally, the sharp lines identified in the 430–520 nm wavelength range are attributed to the intraconfigurational transitions from the ground state to the ^3^P_J_ + ^1^I_6_ multiplets (Figure 10).

### 3.3. Comparative Study of the Synthetic Methods Based on the ^1^D_2_ Emission Lifetime of Y_2_O_3_: Pr^3+^ Nanocrystals

**Figure 12:**
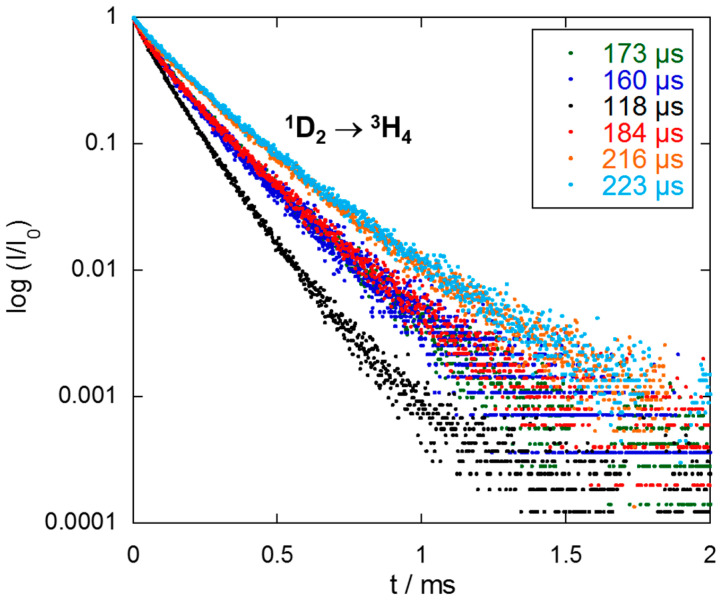
Luminescence decay curves of the Pr^3+ 1^D_2_ → ^3^H_4_ emission of Y_2_O_3_: Pr^3+^ NCs prepared by: combustion method after calcination at 900 °C for 4 h (green); molten salt procedure at 500 °C followed by 10 washing cycles (blue); sol–gel Pechini using citric acid as chelating agent and followed by calcination at 900 °C for 16 h (black); homogeneous precipitation using 0.832 mol of urea and 200 mL of H_2_O during 2 h reaction followed by thermal treatment at 800 °C for 3 h (red); solvothermal method at 220 °C followed by calcination at 1000 °C for 4 h (orange); and solvothermal method at 220 °C followed by calcination at 900 °C for 4 h (cyan).

Luminescence lifetime (τ) measurements were performed to carry out a quantitative comparison of the luminescence efficiency of Y_2_O_3_: Pr^3+^ NCs obtained by the different synthesis methods. The time evolution of ^1^D_2_ → ^3^H_4_ luminescence detected at 629 nm was recorded after direct excitation into ^3^P_J_ excited state at 429 nm for all synthesized samples. All luminescence decay curves were fitted to a double-exponential behavior that can be attributed to the excitation of both sites and the coincidence of peaks from both sites at this wavelength (Figure 12). This fact, together with the probable energy transfer between both sites [15], could be the reason of this bi-exponential behavior. Consequently, the shorter lifetime component could be assigned to Pr^3+^ ions at the C_2_ site, and the longer one, to Pr^3+^ species at the S_6_ site.

Firstly, diverse parameters were optimized for the five different synthesis procedures to maximize the luminescence lifetime. Assuming that all synthesis methods give rise to a similar occupancy of both crystallographic sites, the observed differences in the luminescence lifetime values can be attributed to parameters such as nanocrystal size, Pr^3+^ distribution, crystallinity, and the presence of remaining reagent residues and molecules adsorbed at the NCs surface. In this context, Y_2_O_3_: Pr^3+^ NCs synthesized by combustion method showed an increase in their average lifetime from 114 µs to 173 µs after the thermal treatment at 900 °C (Figure 12 and Figure S30, ESI). Firing the NCs at this temperature promoted not only a better crystallinity, but also the elimination of organic residues assumed to be the most important channel for non-radiative relaxation processes. This effect of remaining impurities on luminescence decay was clearly observed in NCs prepared via molten salt procedure. Indeed, the lifetime increased with the number of washing cycles. NCs that were washed-up 5, 7, and 10 times provided luminescence average lifetimes of 128, 138, and 160 µs, respectively (Figure 12 and Figures S31 and S32, ESI).

Samples prepared via Pechini method were also analyzed. As expected, an increase in calcination time and temperature (Table 2, entries 1–3) had a positive effect on luminescence average lifetime, raising from 104 to 114 and 118 µs (Figure 12 and Figures S33 and S34, ESI), respectively. This may be attributed to the removal of the organic layer and a better crystallinity of the sample. In addition, the use of EDTA as chelating agent produced a slight improvement in lifetime up to 125 µs (Figure S35, ESI), presumably caused by the monodisperse-size character of these NCs (Table 2, entry 4). However, given that high aggregation was also promoted in this case, the enhancement in radiative lifetime was not remarkable enough to consider EDTA as a better chelating agent.

An increase in the luminescence lifetime was observed for Y_2_O_3_: Pr^3+^ NCs synthetized through homogeneous precipitation method. Indeed, NCs prepared with the initial reaction conditions (Table 5, entry 1) presented an average luminescence average lifetime of 152 µs (Figure S36, ESI). The optimization of the different synthesis parameters not only provided better NCs in terms of size, dispersion, and crystallinity, but also improved the luminescence decay rate. For instance, the reduction in the amount of H_2_O to 200 mL (Entry 2) led to an increase in the lifetime up to 161 µs (Figure S37, ESI). In this line, 184 µs as a luminescence lifetime (Figure 12) was observed under the optimized reaction conditions based on the use of 200 mL of deionized H_2_O and 0.832 mol of urea (Entry 3).

Finally, we studied the Y_2_O_3_: Pr^3+^ NCs synthesized via solvothermal method (Table 6), which showed the longest luminescence lifetimes. A luminescence decay time of 198 μs (Figure S38, ESI) was observed for NCs prepared at 180 °C and 800 °C as reaction and calcination temperatures, respectively (Table 6, entry 1). The use of reaction conditions that produce a strong NC aggregation led to a decrease in the lifetime values (Entries 3–5 and Figures S39–S41, ESI).

Conversely, an increase in the reaction and calcination temperatures improves the crystallinity, suppressing the observed organic layer, thus providing NCs with longer values for the luminescence lifetime (Entries 2, 6 and 7, Figure 12, and Figure S42, ESI). A radiative decay of 216 μs (Figure 12) was measured using 220 °C and 1000 °C as reaction and calcination temperatures, respectively (Entry 7). Similarly, a subtle increase in the luminescence lifetime up to 223 μs (Figure 12) was obtained with 220 °C and 900 °C (Entry 6).

**Figure 13:**
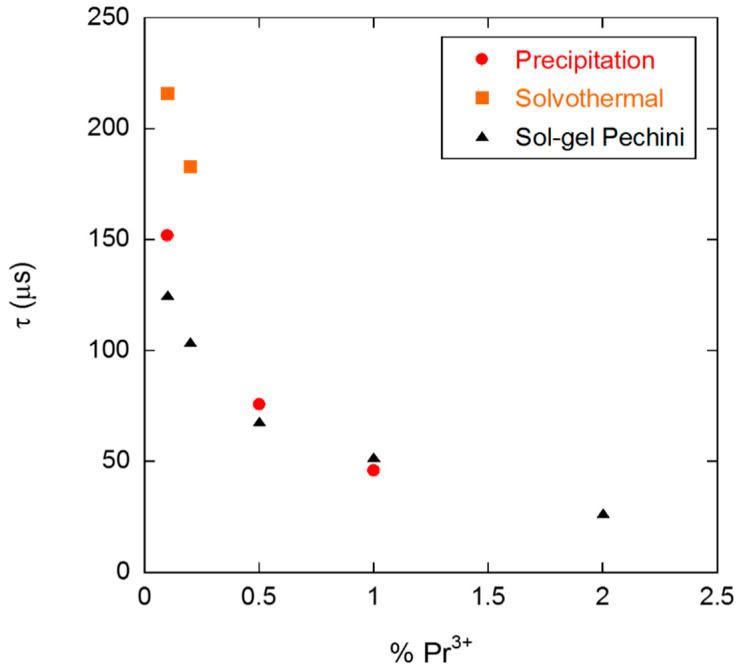
Dependence of the average lifetime on Pr^3+^ concentration for NCs obtained through solvothermal, precipitation, and sol–gel Pechini methods.

The variations of luminescence lifetime attributed to non-radiative processes can be related to factors such as nanocrystal size, annealing temperature, impurities content, trap defects associated with the synthesis method, and small fluctuations in Pr^3+^ concentration, to name a few. In fact, an increase in the nanocrystal size generally led to a reduction of surface effects and thus an improvement of luminescence properties, i.e., an increase of the luminescence lifetime (see Table 5; Table 6, entries 2, 6, and 7). On the other hand, it seems that an increase in the NC aggregation produces a decrease in the lifetime values (see Table 6, entries 3–5). The NCs described in the present work are bigger than 20 nm, and the Pr^3+^ concentration is low (0.1%). These features can minimize the surface effects mainly due to the small number of Pr^3+^ ions located at the surface and the adsorption of molecules from the environment [50,51]. In this line, we have additionally studied the dependence of the emission lifetime on Pr^3+^ concentration for NCs obtained through solvothermal, precipitation, and sol–gel Pechini methods (Figure 13). As expected, a decrease in the luminescence decay rate values with an increase in the Pr^3+^ concentration was observed, thus confirming that 0.1% is the optimal Pr^3+^ content, regardless the synthesis procedure.

All things considered, the optimization of the different synthesis parameters for each discussed method has allowed to improve the optical properties of Y_2_O_3_: Pr^3+^ NCs, therefore achieving longer luminescence lifetimes for the visible red Pr^3+^ emission (^1^D_2_ → ^3^H_4_). Table 7 summarizes the optimized lifetimes for the NCs prepared via the five synthesis procedures. Some of the presented methods have proven to produce NCs with superior optical properties. Among the different procedures, the solvothermal synthesis provided the best results. This method is not only reproducible and time-efficient, but also produces NCs with appropriate size and dispersion. In addition, these Y_2_O_3_: Pr^3+^ NCs showed one of the most intense emission spectra together with the longest luminescence lifetime. While the luminescence lifetime value for bulk 0.1% Pr^3+^-doped Y_2_O_3_ is reported to be 124 µs [32], some authors have described emission decay rates between 110–115 µs for Y_2_O_3_: Pr^3+^ (0.1%) NCs [17,52]. To the best of our knowledge, 180 µs is the longest luminescence emission lifetime reported to date for Pr^3+^-doped yttria NCs [53]. Consequently, it is worth noting that three of the optimized methods described herein provide Y_2_O_3_: Pr^3+^ NCs with the longest luminescence lifetime values described so far.

## 4. Conclusions

In summary, we have successfully implemented five different wet-chemistry methods for the synthesis of Y_2_O_3_: Pr^3+^ NCs. An extensive characterization of these NCs was performed by a wide variety of techniques. The synthesis method has been found to have a crucial effect on both structural and optical properties, namely NC size, dispersion, morphology, and luminescence lifetime. All samples showed a pure cubic phase and good grade of crystallinity, while the NC size and dispersion were dependent on the synthesis procedure. In addition, an in-depth optical characterization was carried out by photoluminescence excitation and emission spectroscopy and luminescence lifetime measurements. In particular, emission from the Pr^3+ 1^D_2_ state at two available crystallographic sites C_2_ and S_6_ was observed for all synthesized Y_2_O_3_: Pr^3+^ NCs, while emission from ^3^P_J_ was completely absent. The reaction conditions were also optimized for each synthesis method in order to obtain the best optical response, measured as the longest luminescence decay time. Finally, a comparative study of the different methods on the basis of the fluorescence average lifetime of so-obtained Y_2_O_3_: Pr^3+^ NCs was performed. In this line, the solvothermal synthesis was demonstrated to be the best-suited method to produce Y_2_O_3_: Pr^3+^ NCs with the most intense luminescence and longest lifetimes (τ = 223 µs).

As a final remark, this work illustrates perfectly how different synthesis methods can be used for the preparation of Y_2_O_3_: Pr^3+^ NCs with amazingly different structural and optical properties. In this context, the present study may help in selecting the most appropriate nanocrystal synthesis route for other oxides depending on the desired structural and optical properties.

## Figures and Tables

**Table 1:** Raman peaks position, width (FWHM) and their symmetry assignment of Y_2_O_3_: Pr^3+^ NCs synthesized by the different methods. ω and FWHM in cm^−1^. In bold the most intense Raman peak.

Symmetry	Combustion		Molten Salt		Pechini		Precipitation		Solvothermal	
	ω	FWHM	ω	FWHM	ω	FWHM	ω	FWHM	ω	FWHM
T_g_	128.3	3.4	130	18.7	128.4	2.9	128.3	3.4	128.6	2.8
A_g_	160.4	3.9	160.4	4.2	160.4	4.2	160	4.3	160.7	3.3
E_g_	192.6	4.4	193.5	2.3	--	--	--	--	192.4	4.6
T_g_	234.1	1.7	--	--	--	--	--	--	234	3.1
T_g_	--	--	288.4	29.8	287.7	19	285.3	16.5	--	--
T_g_	316.2	5.9	315.5	2.8	316.2	5.1	315.8	4.4	316.5	3.8
T_g_ +E_g_	326.0	8.2	329	6	328.7	8.8	328.4	10.3	329.4	6.3
**T_g_**	**376.4**	**6.8**	**376.3**	**6.1**	**376.4**	**5.9**	**376**	**6.5**	**376.5**	**5.3**
A_g_	430	9.7	429.6	5.8	429.9	7.9	429.8	7.6	431.4	9.1
T_g_	468	9.71	468	9	467.54	11.2	467.3	10.6	468.2	9
T_g_	591	17.9	592.1	13.7	590.97	20	--	--	591.6	12.3

**Table 2:** Optimization parameters for the synthesis of Y_2_O_3_: Pr^3+^ NCs by sol–gel Pechini method.

Entry	Chelating Agent	Calcination Time (h)	Calcination T (°C)	Observations
1	Citric acid	16	800	Surrounding organic layer.
2	Citric acid	24	800	Surrounding organic layer.
3	Citric acid	16	900	Well dispersed. 26(6) and 62(16) nm.
4	EDTA	16	900	Aggregated NCs of 40(12) nm.

**Table 3:** Optimization parameters for the synthesis of Y_2_O_3_: Pr^3+^ NCs by homogeneous precipitation method.

Entry	Time (h)	V H_2_O (mL)	Urea Amount (mol)	Observations
1	120	360	0.485	Well-dispersed spheres of 260(26) nm; NC size (PXRD): 20.2(2) nm.
2	120	720	0.485	Well-dispersed spheres of 233(41) nm; NC size (PXRD): 21.8(3) nm.
3	120	200	0.485	Well-dispersed spheres of 229(20) nm; NC size (PXRD): 25.2(4) nm.
4	120	360	0.166	Very well-dispersed spheres of 338(35) nm; NC size (PXRD): 26.9(3) nm.
5	120	360	1.415	Agglomerated spheres of 75(34) nm; NC size (PXRD): 22.2(2) nm.
6	120	360	0.832	Well-dispersed spheres of 219(24) nm; NC size (PXRD): 21.2(2) nm.
7	120	200	0.832	Very well-dispersed spheres of 155(20) nm; NC size (PXRD): 30(5) nm.

**Table 4:** Optimization parameters for the synthesis of Y_2_O_3_: Pr^3+^ NCs by solvothermal method.

Entry	RE (mmol)	Alcohol	Reaction T (°C)	Calcination T (°C)/Time (h)	Observations
1	7.8478	EG	180	800/4	Some aggregation; 31(7) nm (TEM), 21.1(1) nm (PXRD); organic layer
2	7.8478	EG	180	900/4	Slightly aggregated NCs; 42(11) nm (TEM), 31.1(3) nm (PXRD).
3	15.6957	EG	180	900/4	Strong aggregation; 40(10) nm (TEM), 43.1(3) nm (PXRD).
4	7.8478	EtOH	180	900/4	Strong aggregation; 28(10) nm (TEM).
5	7.8478	EG	180	900/8	Strong aggregation
6	7.8478	EG	220	900/4	Slightly aggregated NCs; 47(12) nm (TEM).
7	7.8478	EG	220	1000/4	Slightly aggregated NCs; 68(15) nm (TEM), 52.8(5) nm (PXRD).

**Table 5:** Luminescence lifetime of the Pr^3+ 1^D_2_ → ^3^H_4_ emission transition at RT for selected Y_2_O_3_: Pr^3+^ NCs synthesized by homogeneous precipitation method.

Entry	V H_2_O (mL)	Urea Amount (mol)	Observations	τ (^1^D_2_)/µs
1	360	0.485	Well-dispersed spheres of 260(26) nm; NC size: 20.2(2) nm.	152
2	200	0.485	Well-dispersed spheres of 229(20) nm; NC size: 25.2(4) nm.	161
3	200	0.832	Very well-dispersed spheres of 155(20) nm; NC size: 30(5) nm.	184

**Table 6:** Luminescence lifetime of the Pr^3+ 1^D_2_ → ^3^H_4_ emission transition at RT for Y_2_O_3_: Pr^3+^ NCs synthetized by solvothermal method ^a^.

Entry	Alcohol	T (°C)	Calcination T (°C)/Time (h)	Observations	τ (^1^D_2_)/µs
1	EG	180	800/4	Aggregation; 31(7) nm (TEM), 21(1) nm (PXRD); organic layer.	198
2	EG	180	900/4	Slight aggregation; 42(11) nm (TEM), 31.1(3) nm (PXRD).	202
3 ^b^	EG	180	900/4	Strong aggregation; 40(10) nm (TEM), 43.1(3) nm (PXRD).	144
4	EtOH	180	900/4	Strong aggregation; 28(10) nm.	163
5	EG	180	900/8	Strong aggregation.	135
6	EG	220	900/4	Slight aggregation; 47(12) nm.	223
7	EG	220	1000/4	Slight aggregation; 68(15) nm (TEM), 52.8(5) nm (PXRD).	216

^a^ 7.848 mmol RE. ^b^ 15.696 mmol RE.

**Table 7:** ^1^D_2_ → ^3^H_4_ Pr^3+^ average lifetimes of Y_2_O_3_: Pr^3+^ NCs after optimization of the different synthesis methods.

Synthesis Method	τ (^1^D_2_)/µs
Combustion	173
Molten salt	160
Pechini	118
Precipitation	184
Solvothermal	223

## Supplementary Materials

The following are available online at https://www.mdpi.com/2079-4991/10/8/1574/s1, Figure S1: PXRD analysis, Figure S2: Raman spectra, Figure S3: Comparison of the most prominent Raman peak of Y_2_O_3_: Pr^3+^ NCs, Figure S4: Reflectance spectra, Figure S5: TG + DSC analysis, Figures S6–S25: TEM images, Figure S26: Emission spectra from C_2_ site, Figure S27: Emission spectra from S_6_ site, Figure S28: Excitation spectra at C_2_ site, Figure S29: Excitation spectra at S_6_ site, Figures S30–S42: Luminescence lifetime measurements.
