# Supplementary material for: A Comparative Study on Luminescence Properties of Y2O3: Pr3+ Nanocrystals Prepared by Different Synthesis Methods
Source: Nanomaterials (Basel). 2020 Aug 11;10(8):1574. doi: 10.3390/nano10081574 (PMC7466516; doi:10.3390/nano10081574)
Supplement: Supplementary file 1 [file nanomaterials-10-01574-s001.pdf]

SUPPORTING INFORMATION

A Comparative Study on Luminescence Properties of Y_2O_3: Pr^{3+} Nanocrystals Prepared by Different Synthesis Methods

Contents

1. **Powder X-ray Diffraction (PXRD) Analysis…………………………………………....... S2**
2. **Raman Spectroscopy……………………………........…………………................... S3**
3. **Reflectance Spectroscopy………………………………………………......…………... S5**
4. **Thermogravimetric Analysis (TGA+DSC) …........………………...…………..……. S6**
5. **Transmission Electron Microscopy (TEM) Images ……………………….……..… S7**
6. **Fluorescence Emission Spectroscopy…..……….………………….………………... S18**
7. **Fluorescence Excitation Spectroscopy…………..…………………………….…… S20**
8. **Luminescence Lifetime (τ)………………...………………………………………… S22**

1. Powder X-Ray Diffraction (PXRD) Analysis

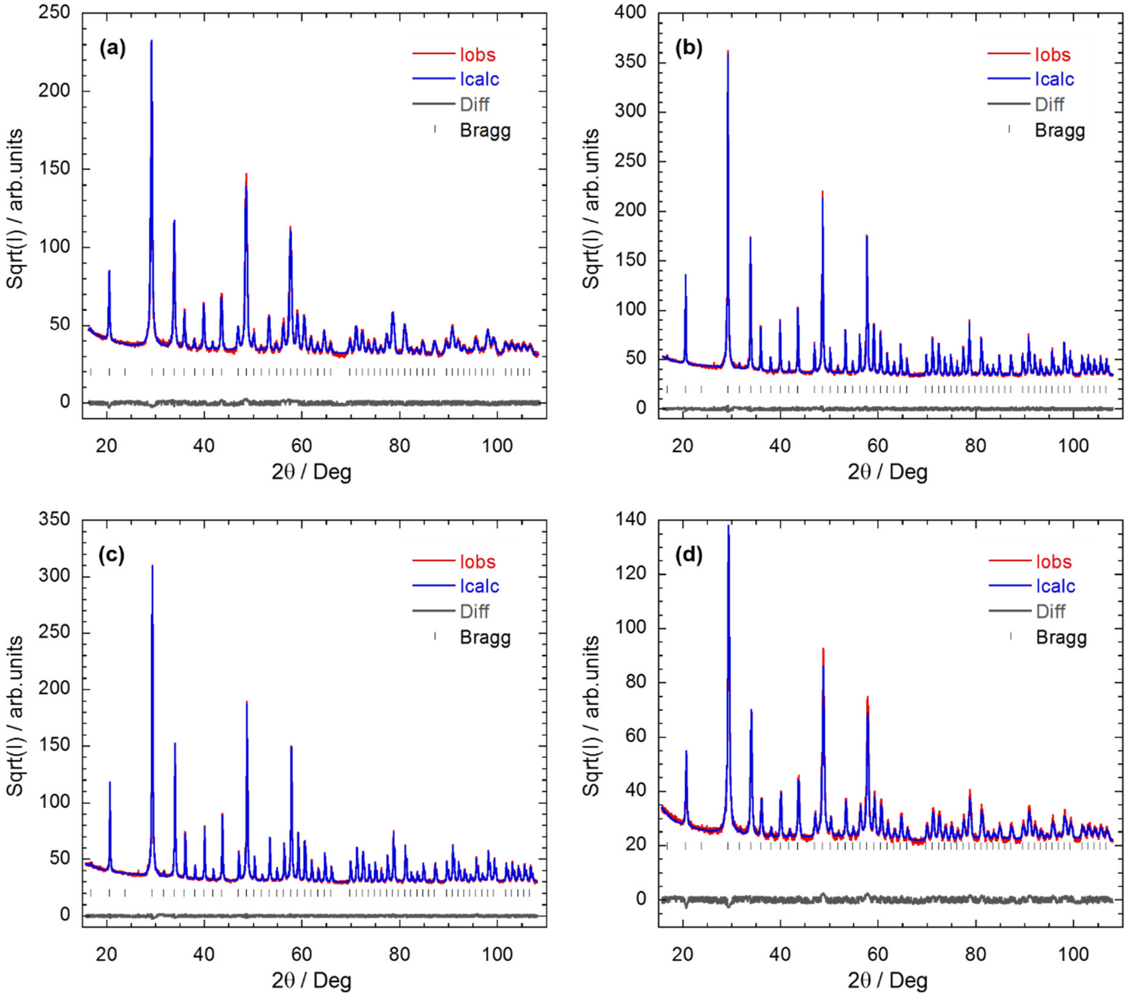

Figure S1. XRD patterns of Y_2O_3: Pr^{3+} NCs obtained by (**a**) combustion method after calcination at 900 ºC for 4 h; (**b**) molten salt method at 500 ºC followed by 10 washing cycles; (**c**) sol-gel Pechini method using citric acid as chelating agent and followed by thermal treatment at 900 ºC for 16 h; and (**d**) homogeneous precipitation using 0.832 mol of urea and 200 ml H_2O during 2 h reaction followed by thermal treatment at 800 ºC for 3 h.

2. Raman Spectroscopy

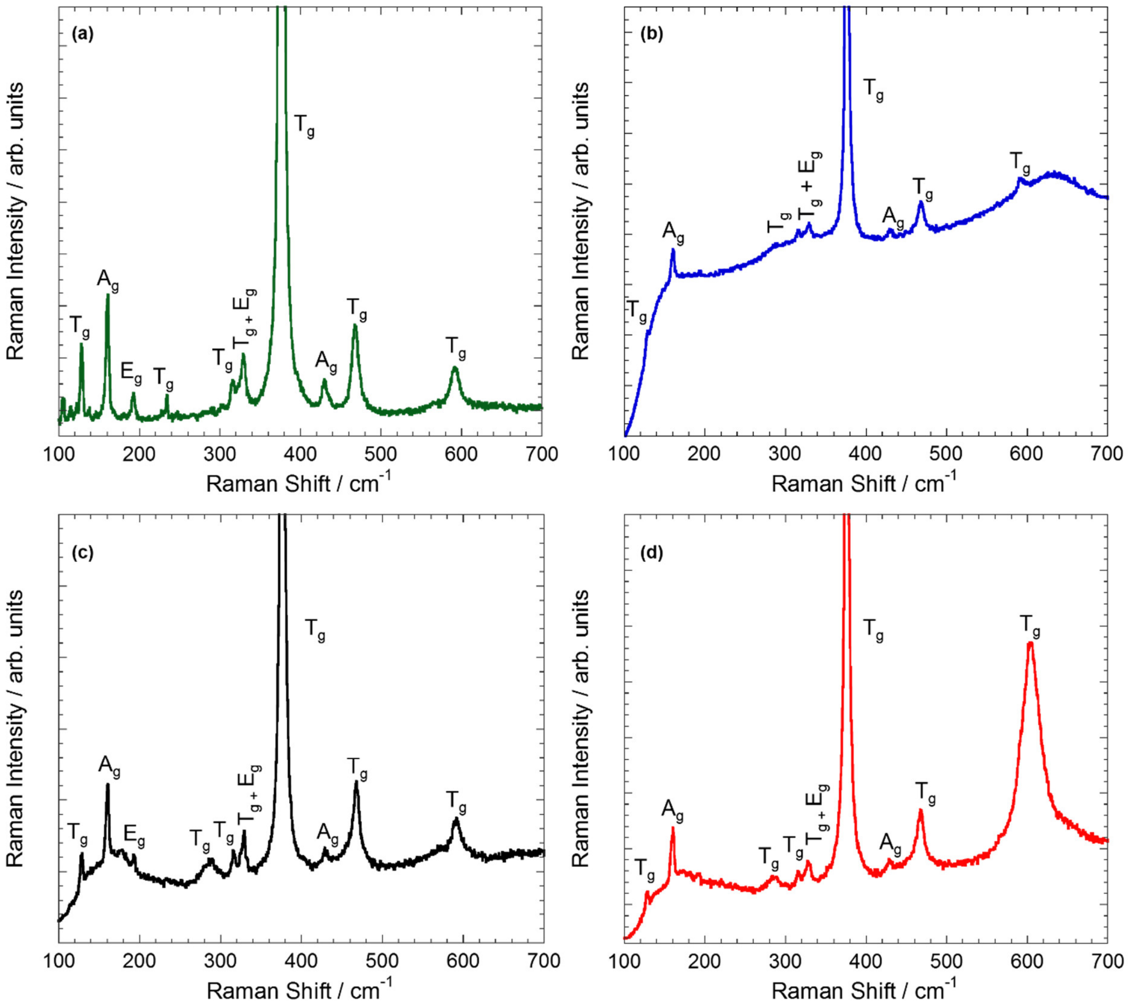

Figure S2. Raman spectra of Y_2O_3: Pr^{3+} NCs obtained by (**a**) combustion method after calcination at 900 ºC for 4 h; (**b**) molten salt method at 500 ºC followed by 10 washing cycles; (**c**) sol-gel Pechini method using citric acid as chelating agent and followed by thermal treatment at 900 ºC for 16 h; and (**d**) homogeneous precipitation method using 0.832 mol of urea and 200 ml of deionized water during 2 h reaction followed by thermal treatment at 800 ºC for 3 h.

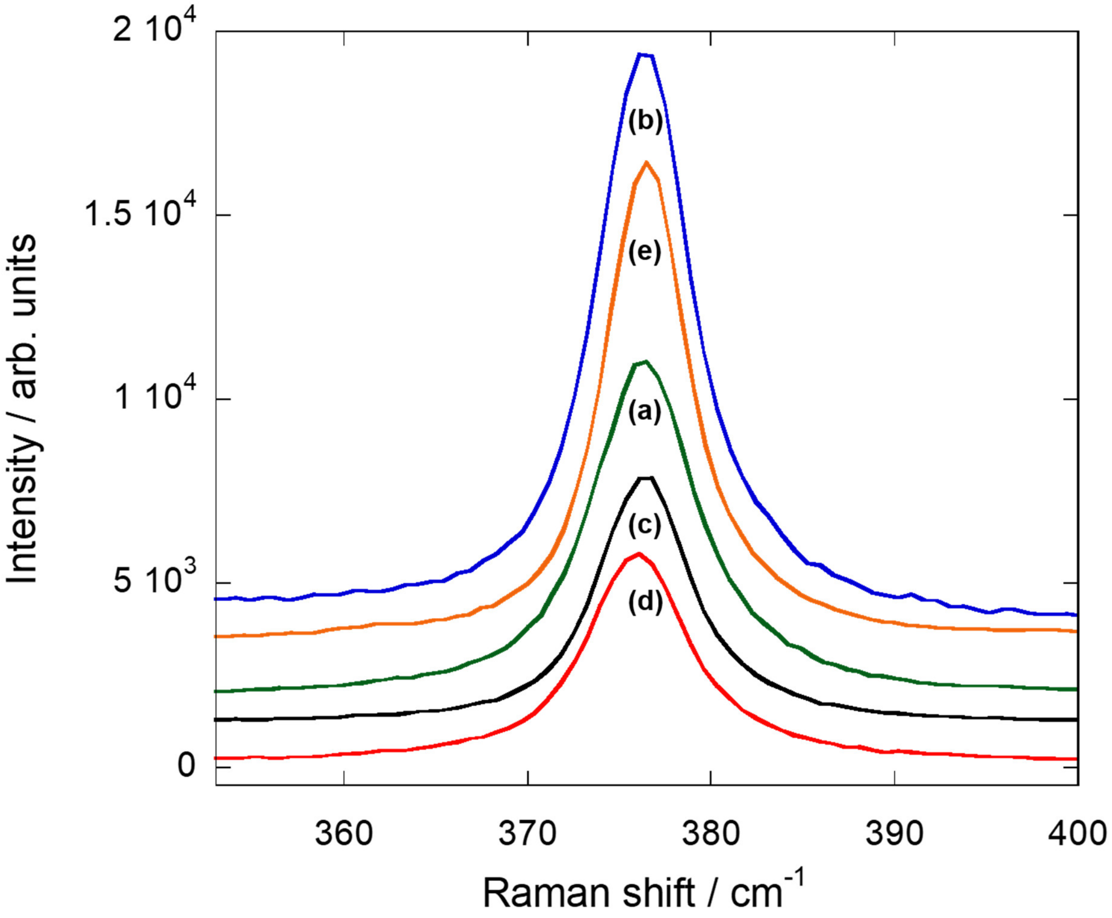

Figure S3. Comparison of the most prominent Raman peak of Y_2O_3: Pr^{3+} NCs prepared by (**a**) combustion method after calcination at 900 ºC for 4h; (**b**) molten salt method at 500 ºC followed by 10 washing cycles; (**c**) sol-gel Pechini method using citric acid as chelating agent and followed by thermal treatment at 900 ºC for 16 h; (**d**) homogeneous precipitation method using 0.832 mol of urea and 200 ml of water during 2 h reaction followed by thermal treatment at 800 ºC for 3 h; and (**e**) solvothermal method at 220 ºC followed by calcination at 1000 ºC for 4 h.

3. Reflectance Spectroscopy

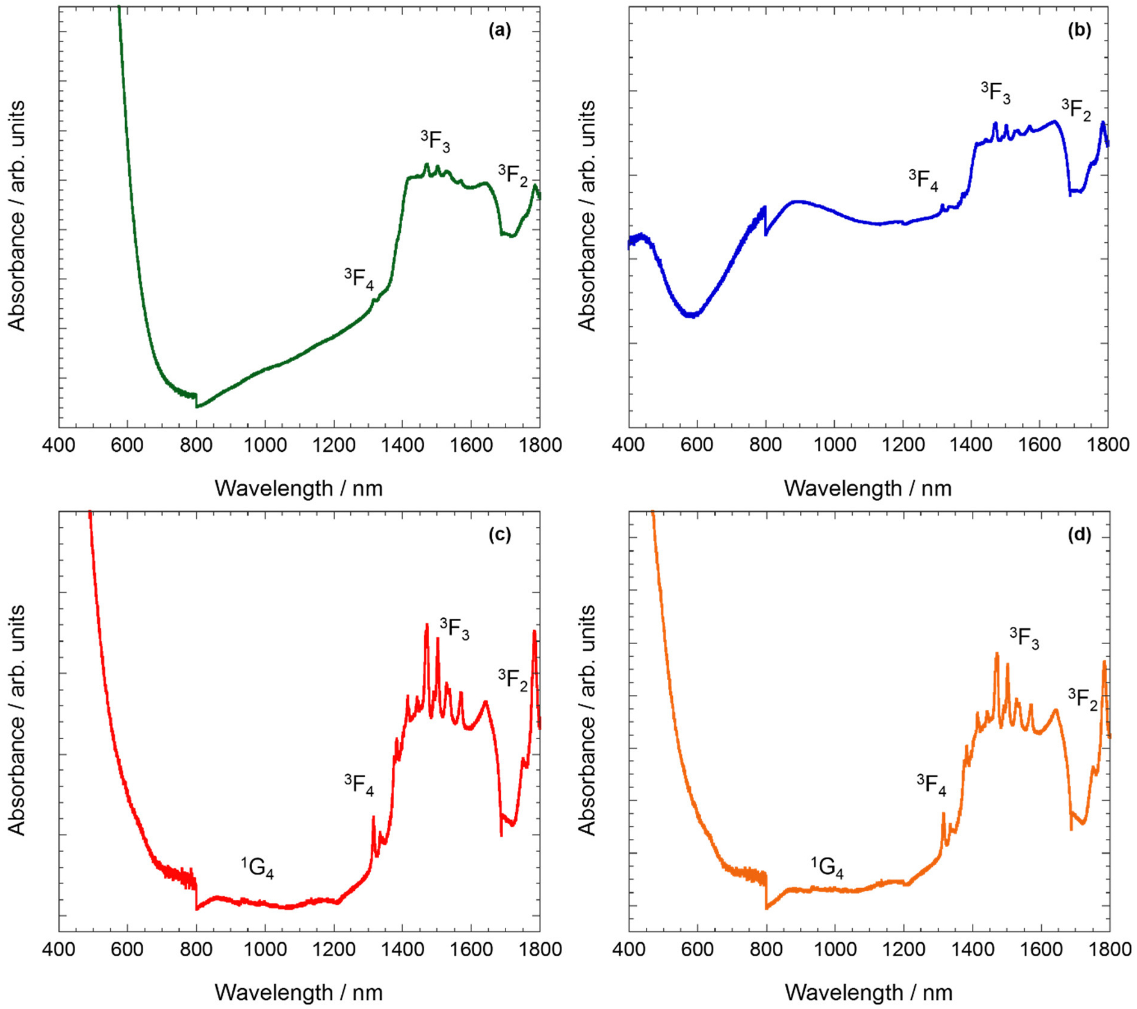

Figure S4. Absorption spectra of Y_2O_3: Pr^{3+} NCs obtained by (**a**) combustion method after calcination at 900 ºC for 4 h; (**b**) molten salt method at 500 ºC followed by 10 washing cycles; (**c**) homogeneous precipitation using urea (0.832 mol) and H_2O (200 mL) during 2 h reaction followed by calcination at 800 ºC (3 h); and (**d**) solvothermal method at 220 ºC followed by calcination at 1000 ºC for 4 h.

4. Thermogravimetric Analysis (TG + DSC)

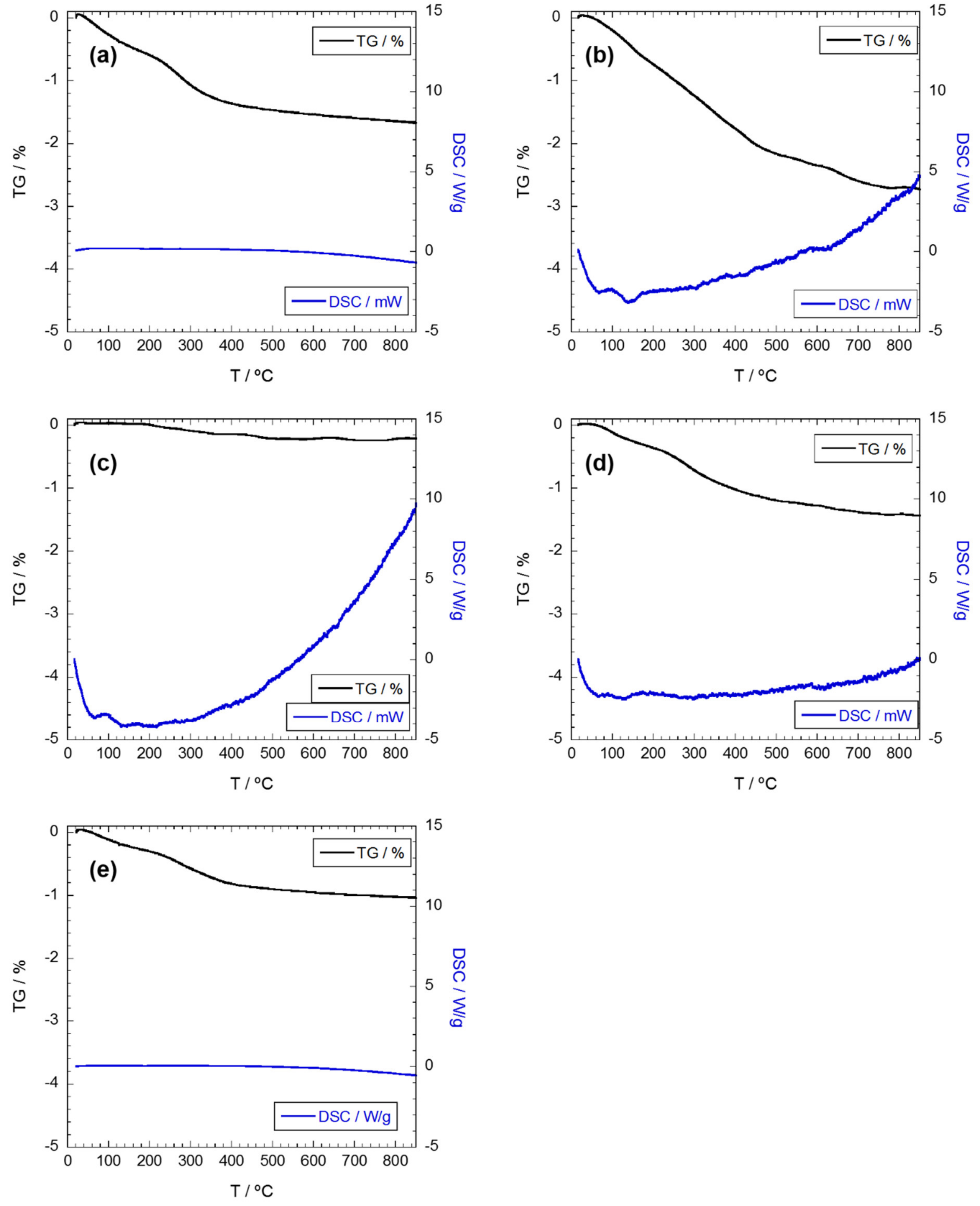

Figure S5. TG + DSC plots of Y_2O_3: Pr^{3+} NCs prepared by (**a**) combustion method after calcination at 900 ºC for 4h; (**b**) molten salt method at 500 ºC followed by 10 washing cycles; (**c**) sol-gel Pechini method using citric acid as chelating agent and followed by thermal treatment at 900 ºC for 16 h; (**d**) homogeneous precipitation method using 0.832 mol of urea and 200 ml of water during 2 h reaction followed by thermal treatment at 800 ºC for 3 h; and (**e**) solvothermal method at 220 ºC followed by calcination at 1000 ºC for 4 h.

5. **Transmission Electron Microscopy (TEM) Images**

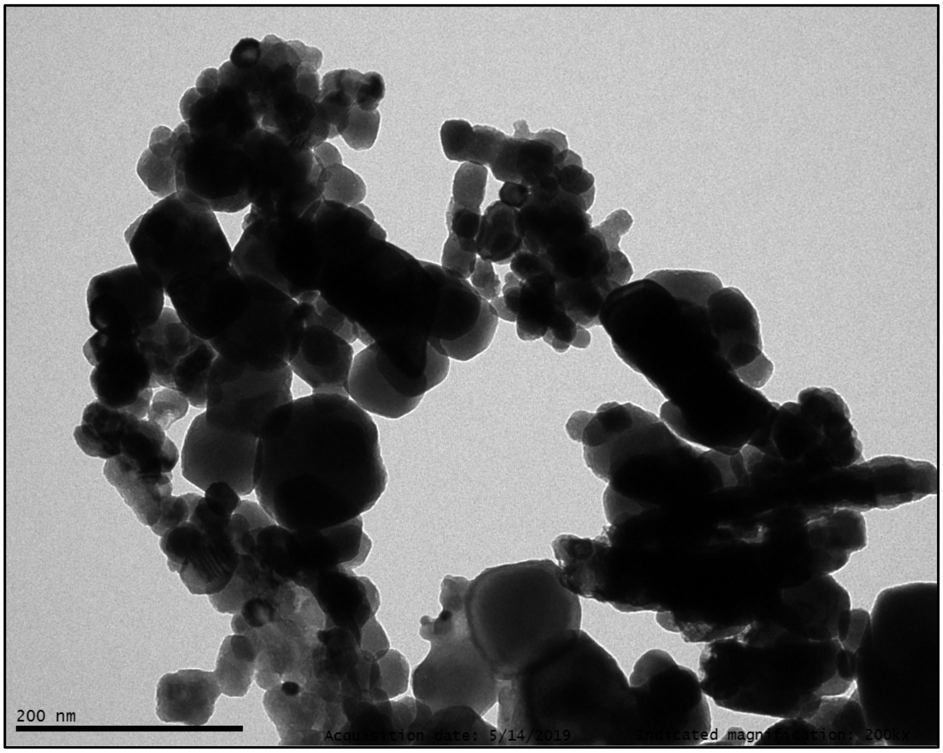

Figure S6. TEM images of Y_2O_3: Pr^{3+} NCs obtained by molten salt method at 500 ºC followed by 10 washing cycles.

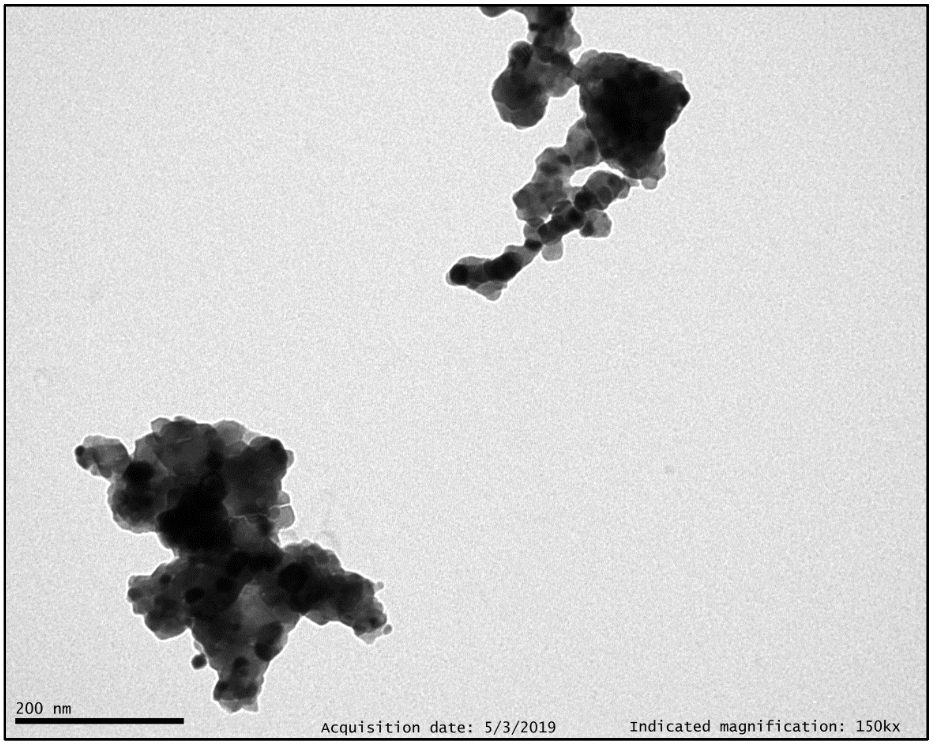

Figure S7. TEM image of Y_2O_3: Pr^{3+} NCs obtained by sol-gel Pechini method using citric acid as chelating agent and followed by thermal treatment at 800 ºC for 16 h.

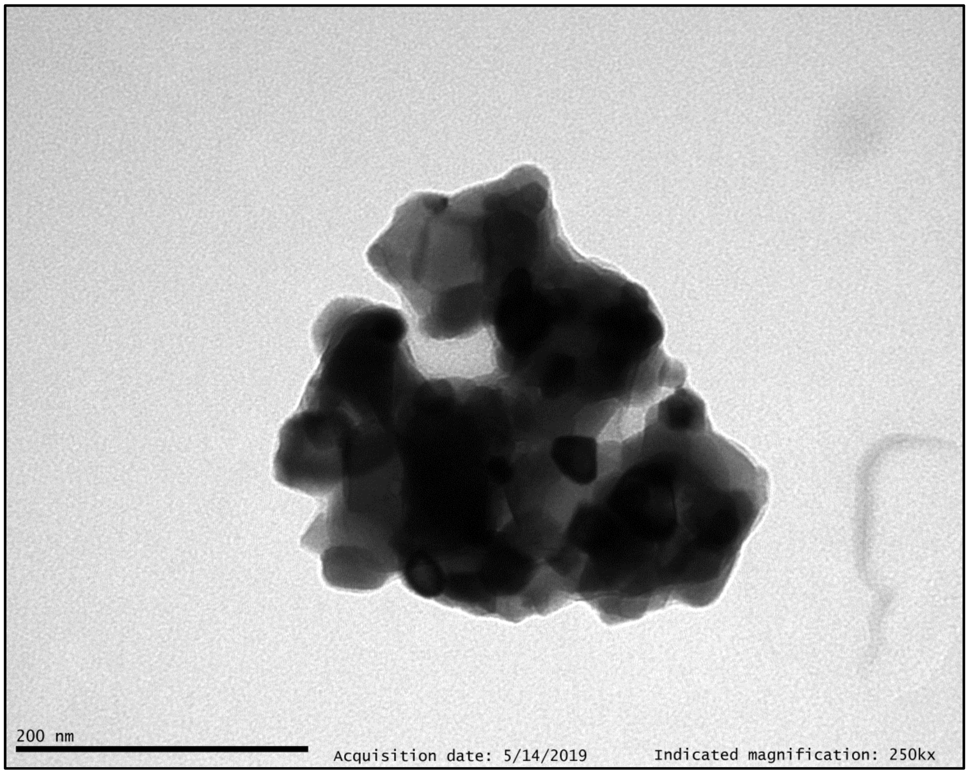

Figure S8. TEM image of Y_2O_3: Pr^{3+} NCs obtained by sol-gel Pechini method using citric acid as chelating agent and followed by thermal treatment at 800 ºC for 24 h.

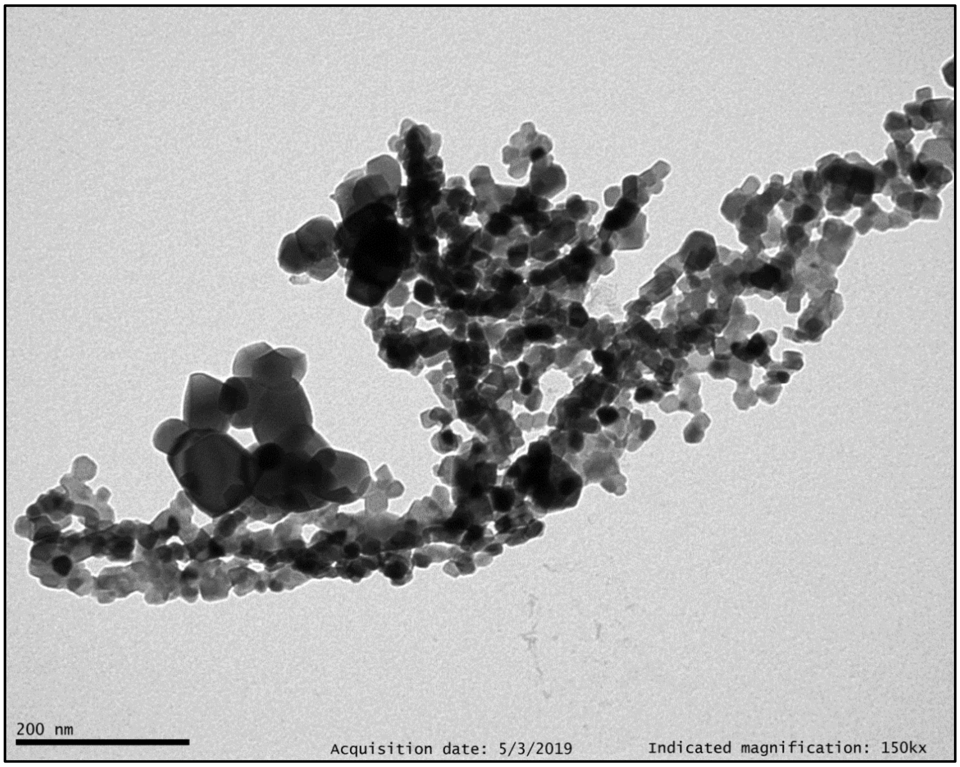

Figures S9. TEM images of Y_2O_3: Pr^{3+} NCs obtained by sol-gel Pechini method using citric acid as chelating agent and followed by thermal treatment at 900 ºC for 16 h.

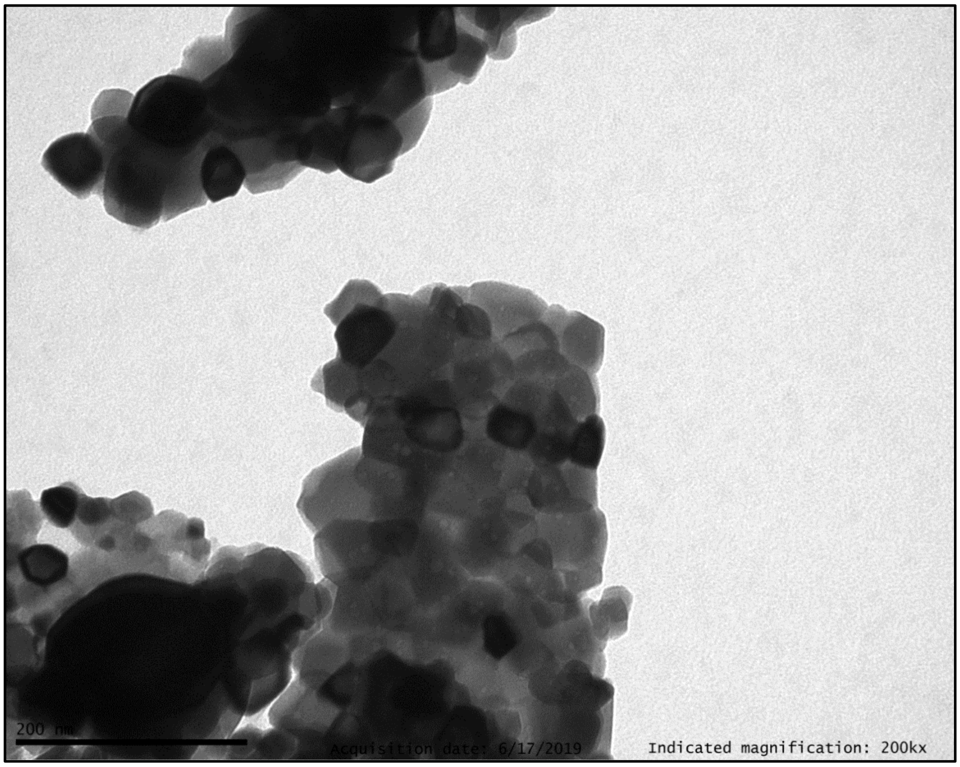

Figure S10. TEM image of Y_2O_3: Pr^{3+} NCs obtained by sol-gel Pechini method using EDTA as chelating agent and followed by thermal treatment at 900 ºC for 16 h.

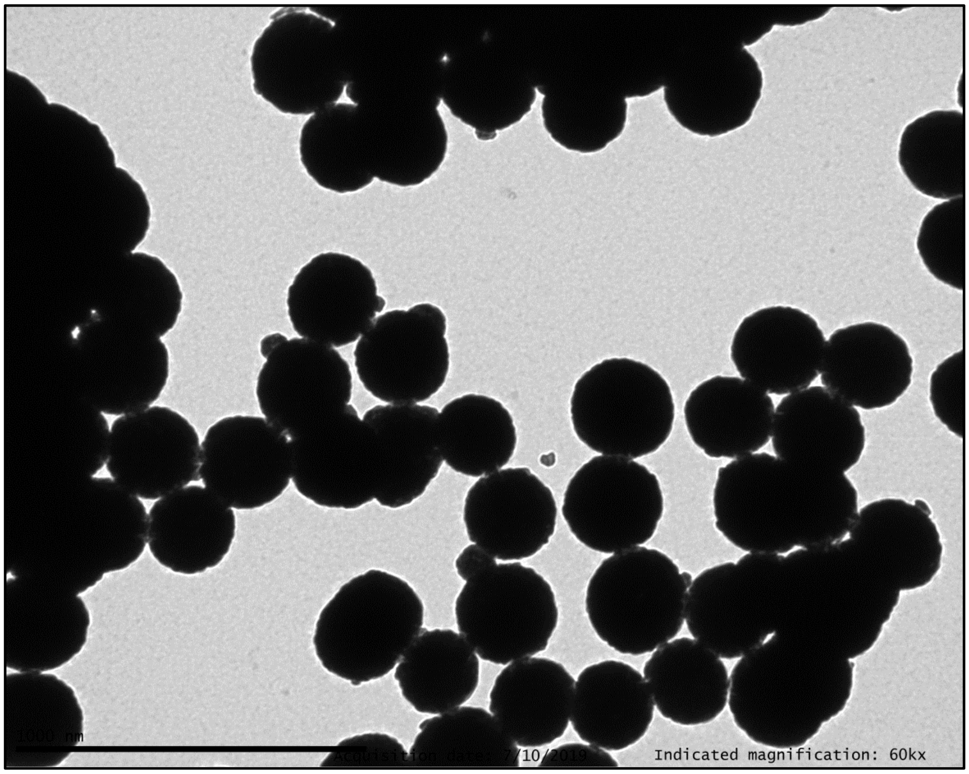

Figure S11. TEM image of Y_2O_3: Pr^{3+} NCs obtained by homogeneous precipitation using 0.485 mol of urea and 360 ml of water during 2 h reaction followed by thermal treatment at 800 ºC for 3 h.

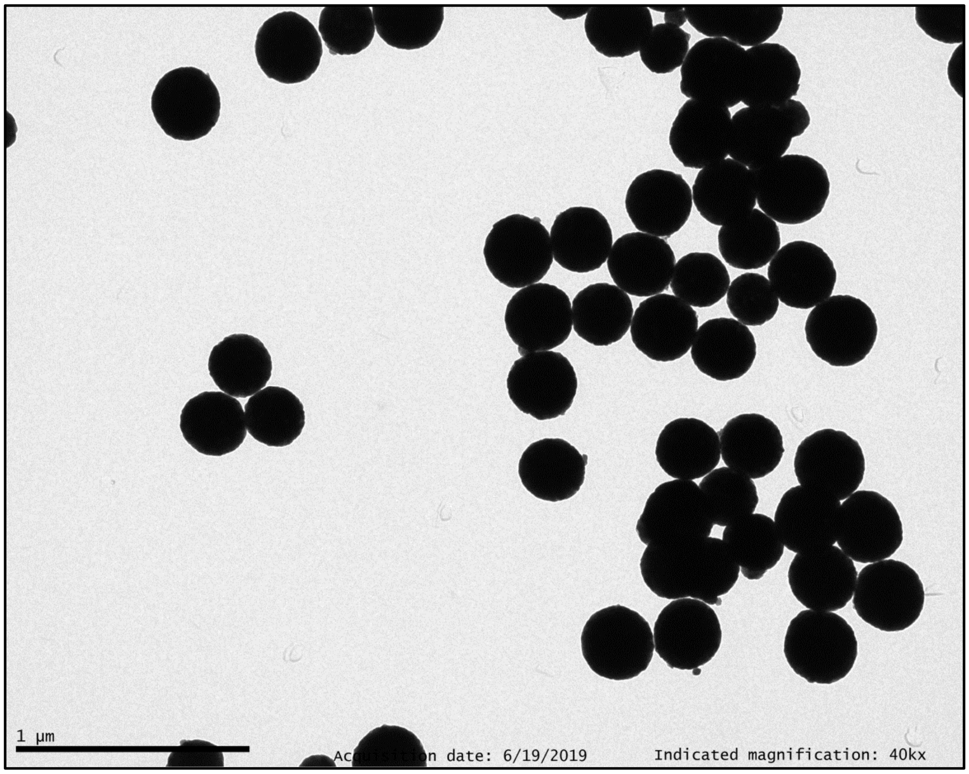

Figure S12. TEM image of Y_2O_3: Pr^{3+} NCs obtained by homogeneous precipitation using 0.485 mol of urea and 720 ml of water during 2 h reaction followed by thermal treatment at 800 ºC for 3 h.

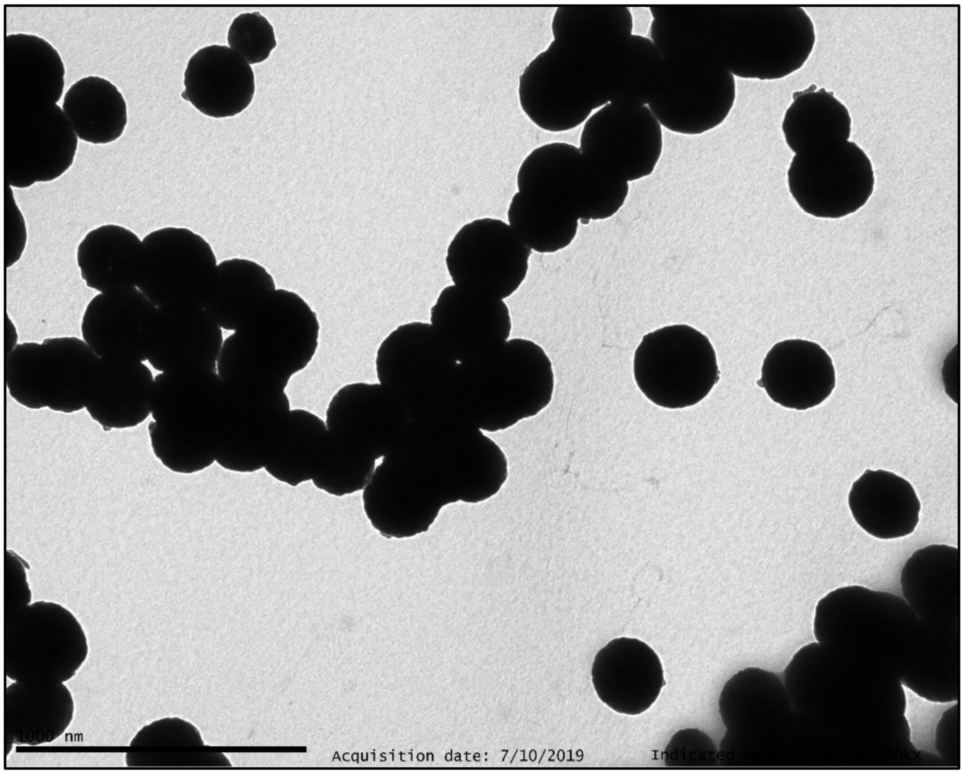

Figure S13. TEM image of Y_2O_3: Pr^{3+} NCs obtained by homogeneous precipitation using 0.485 mol of urea and 200 ml of water during 2 h reaction followed by thermal treatment at 800 ºC for 3 h.

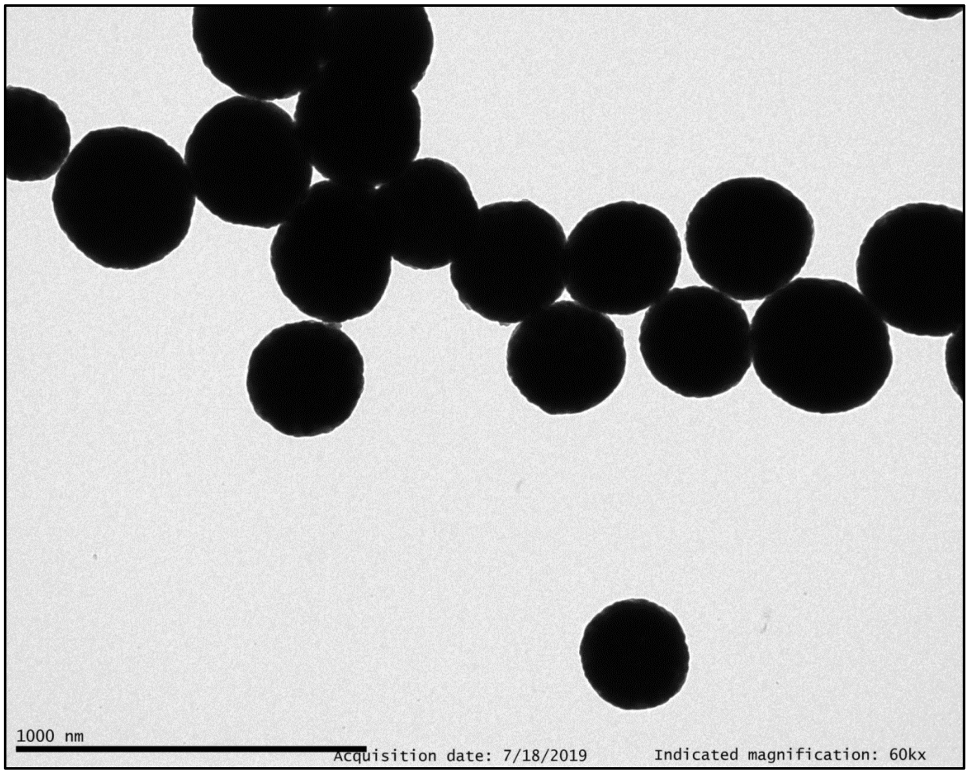

Figure S14. TEM image of Y_2O_3: Pr^{3+} NCs obtained by homogeneous precipitation using 0.166 mol of urea and 360 ml of water during 2 h reaction followed by thermal treatment at 800 ºC for 3 h.

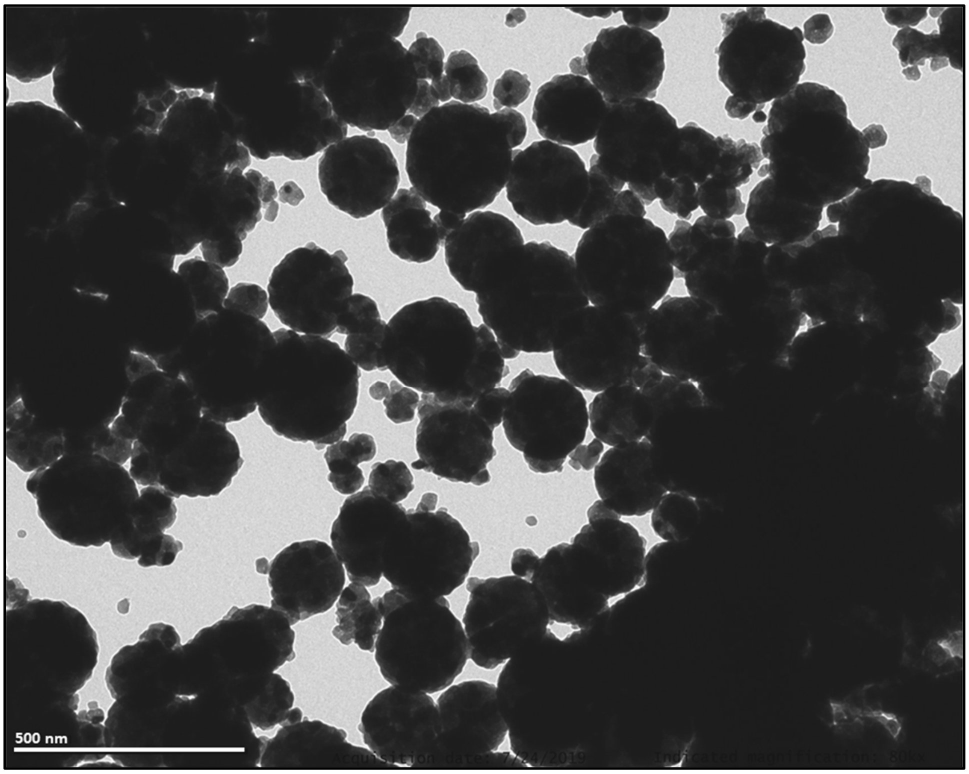

Figure S15. TEM image of Y_2O_3: Pr^{3+} NCs obtained by homogeneous precipitation using 1.415 mol of urea and 360 ml of water during 2 h reaction followed by thermal treatment at 800 ºC for 3 h.

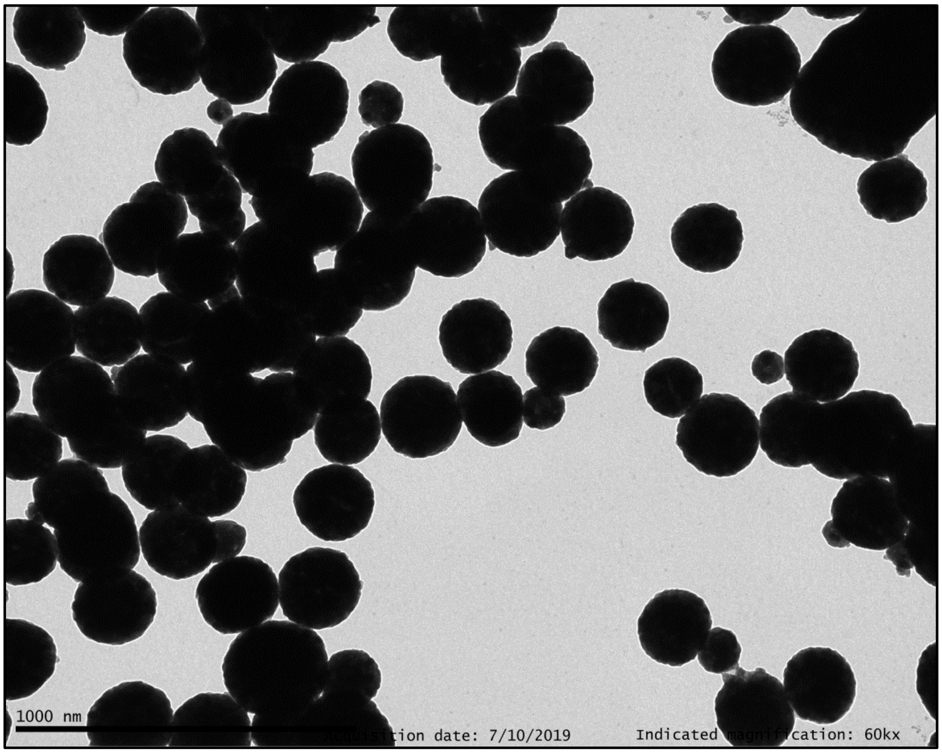

Figure S16. TEM image of Y_2O_3: Pr^{3+} NCs obtained by homogeneous precipitation using 0.832 mol of urea and 360 ml of water during 2 h reaction followed by thermal treatment at 800 ºC for 3 h.

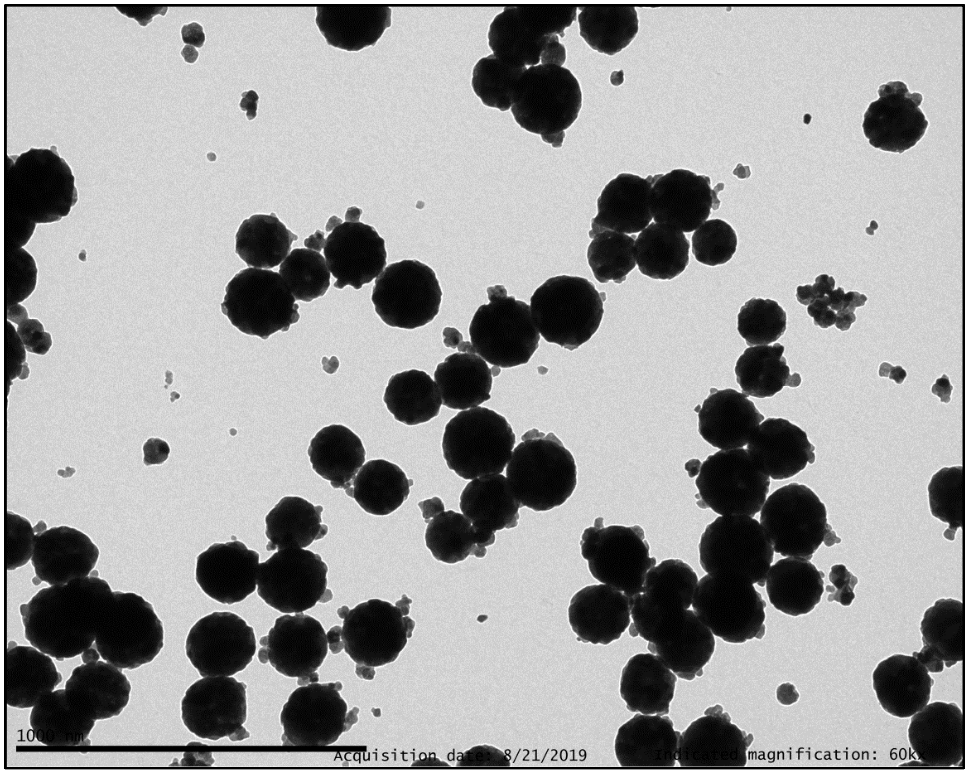

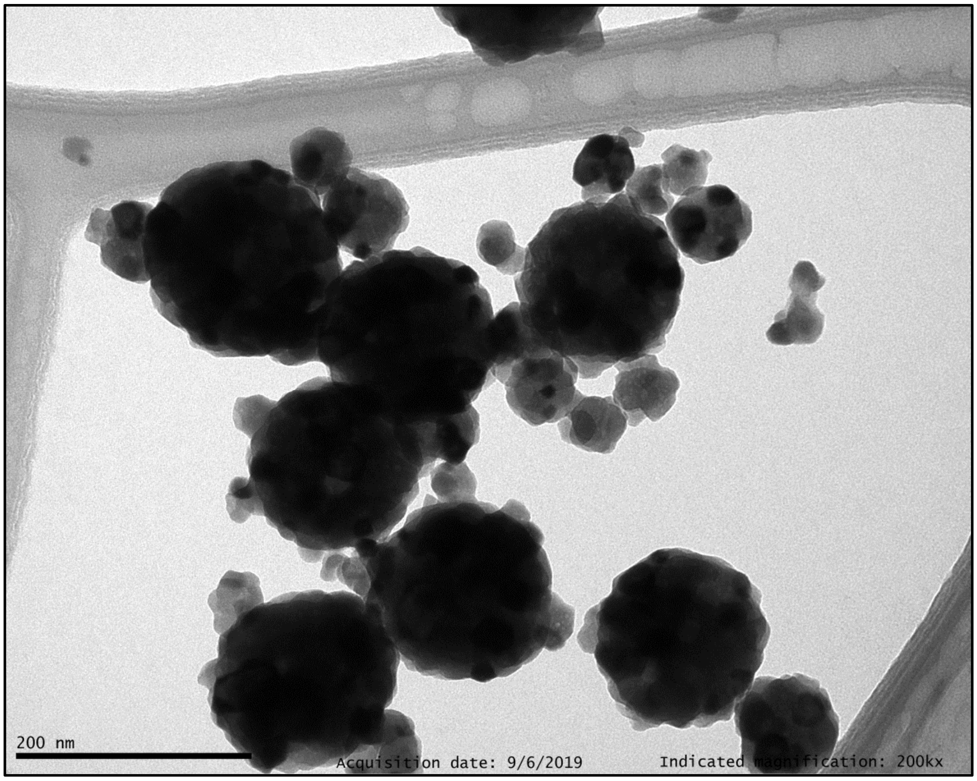

Figures S17 and S18. TEM images of Y_2O_3: Pr^{3+} NCs obtained by homogeneous precipitation using 0.832 mol of urea and 200 ml of water during 2 h reaction followed by thermal treatment at 800 ºC for 3 h.

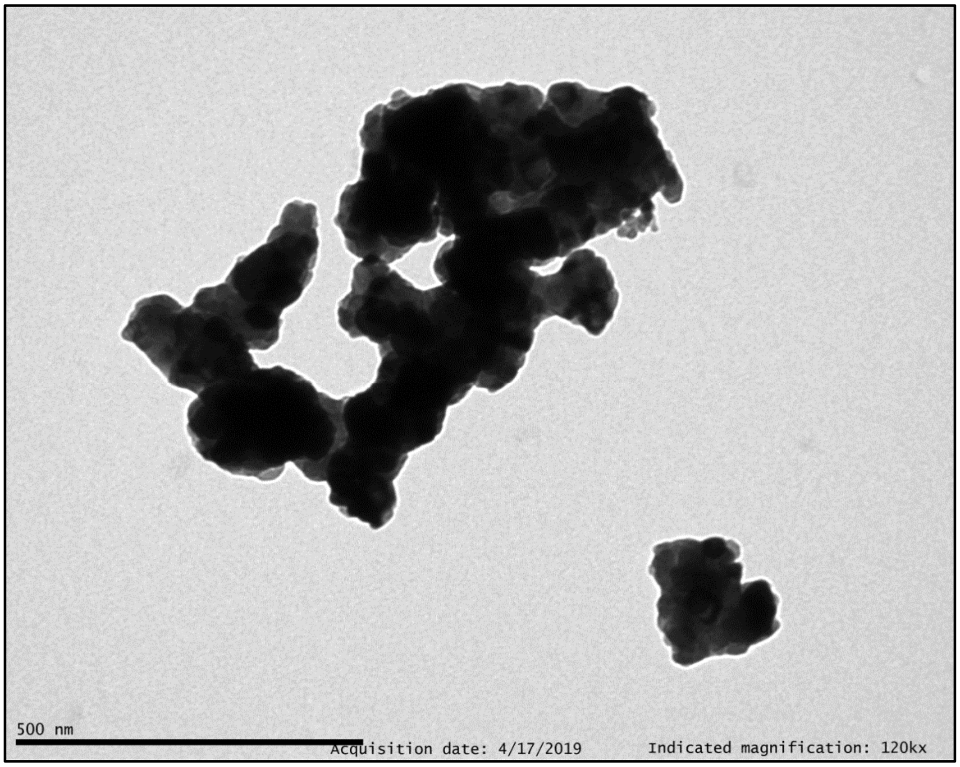

Figure S19. TEM image of Y_2O_3: Pr^{3+} NCs prepared by solvothermal method at 180 ºC for 24 h followed by calcination at 800 ºC for 4 h.

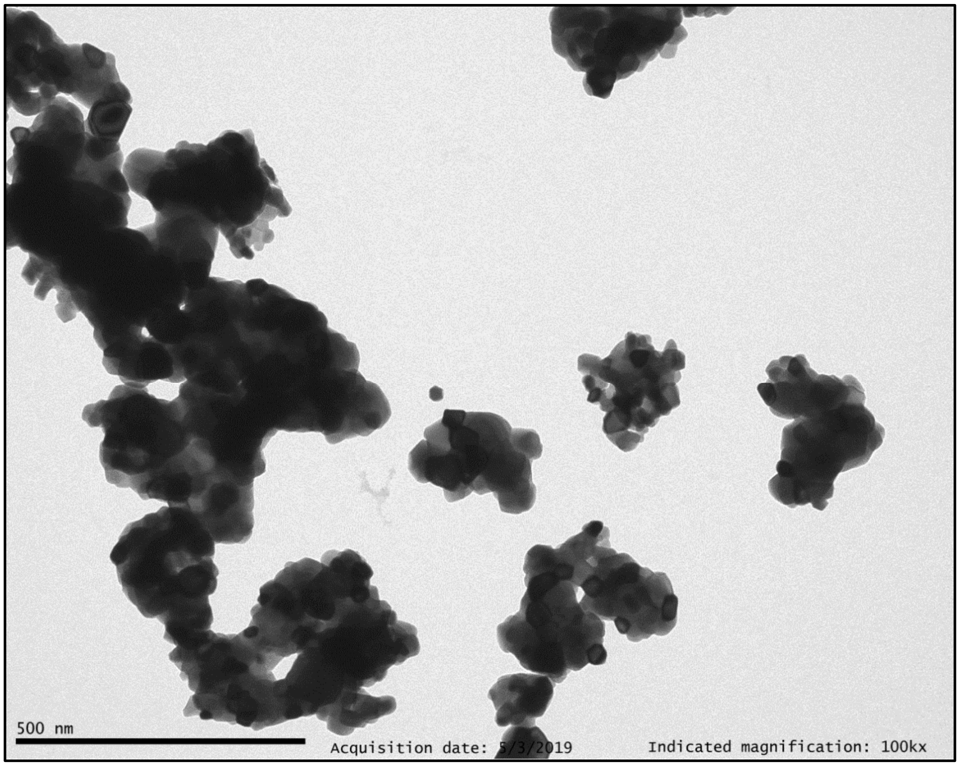

Figure S20. TEM image of Y_2O_3: Pr^{3+} NCs prepared by solvothermal method at 180 ºC for 24 h followed by calcination at 900 ºC for 4 h.

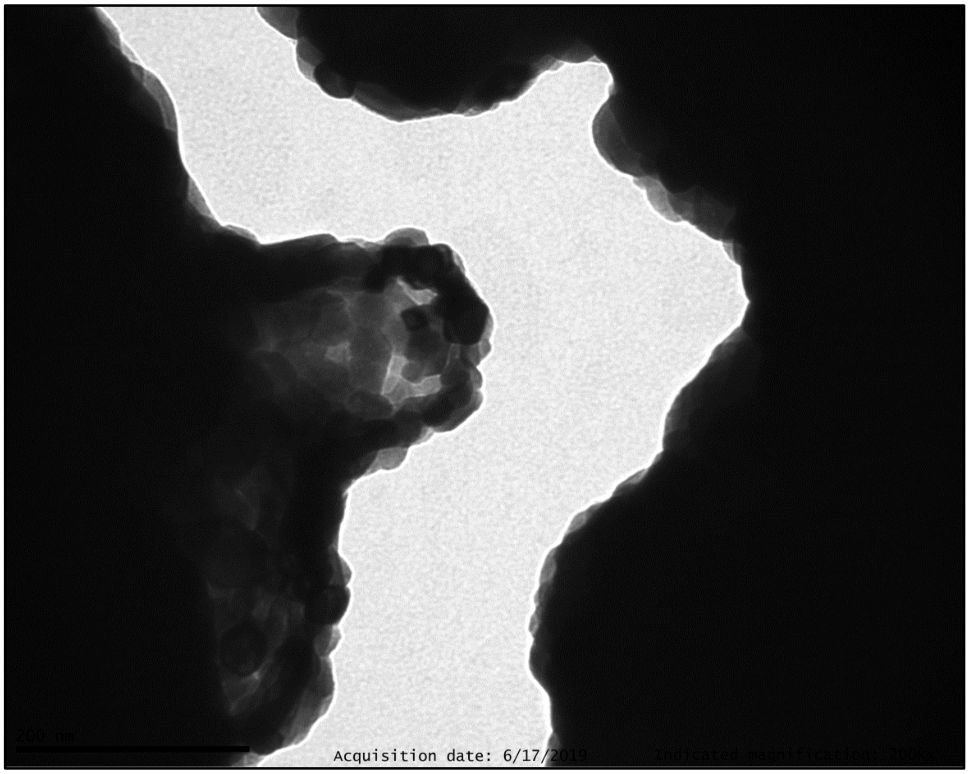

Figure S21. TEM image of Y_2O_3: Pr^{3+} NCs prepared by solvothermal method at 180 ºC for 24 h using a two-fold RE precursor concentration, followed by calcination at 900 ºC for 4 h.

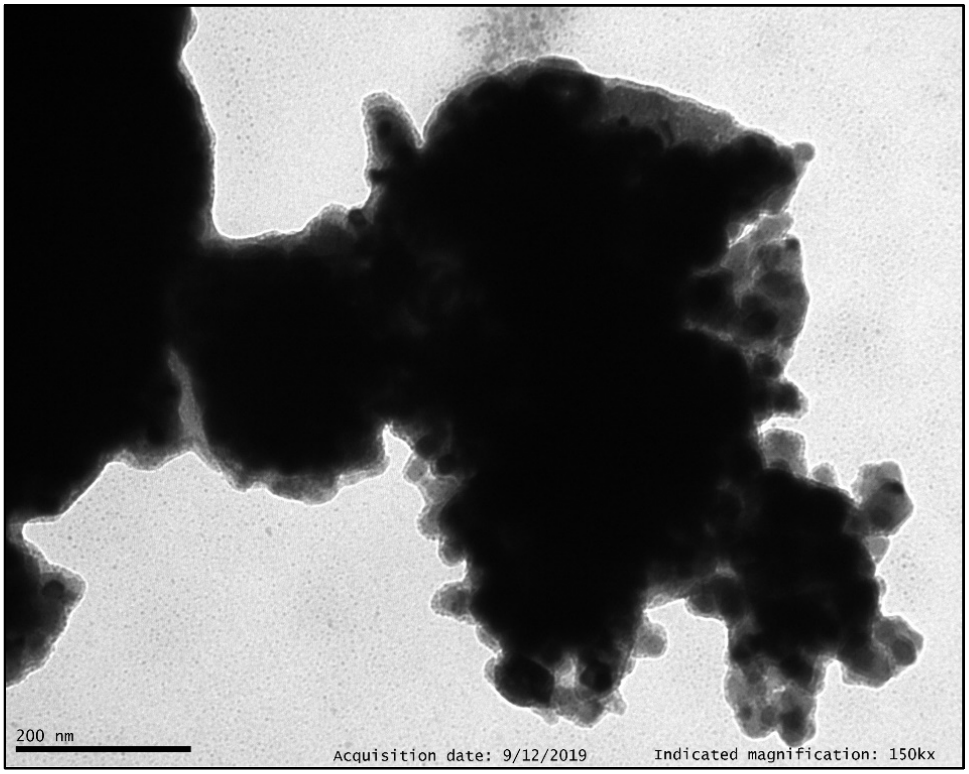

Figure S22. TEM image of Y_2O_3: Pr^{3+} NCs prepared by solvothermal method at 180 ºC for 24 h using EtOH, followed by calcination at 900 ºC for 4 h.

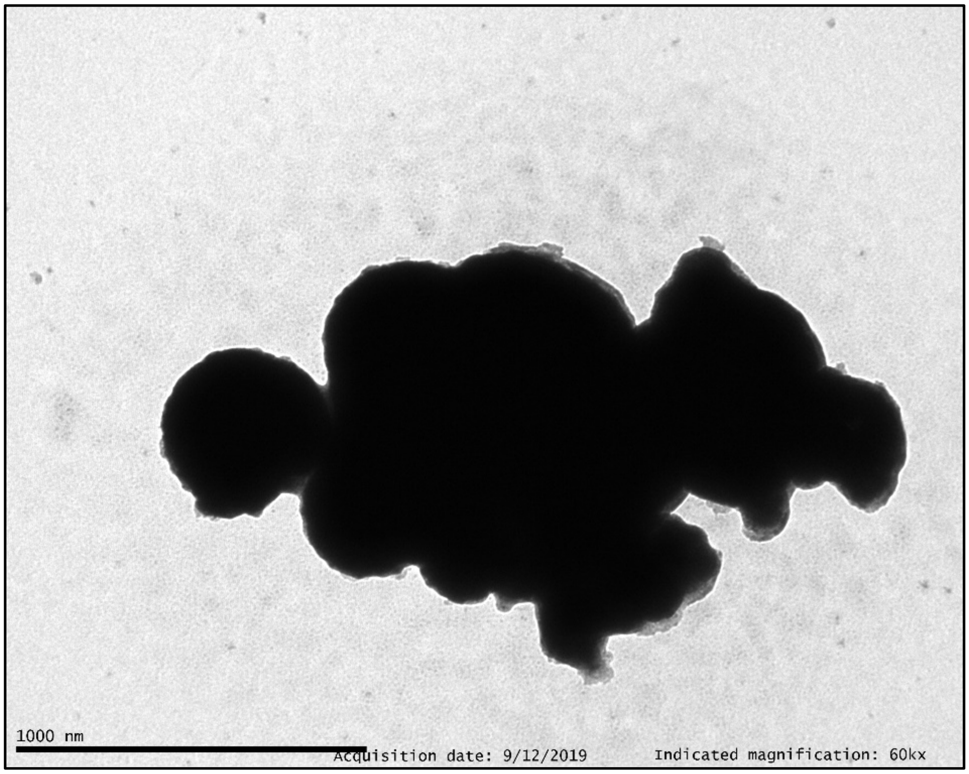

Figure S23. TEM image of Y_2O_3: Pr^{3+} NCs prepared by solvothermal method at 180 ºC for 24 h followed by calcination at 900 ºC for 8 h.

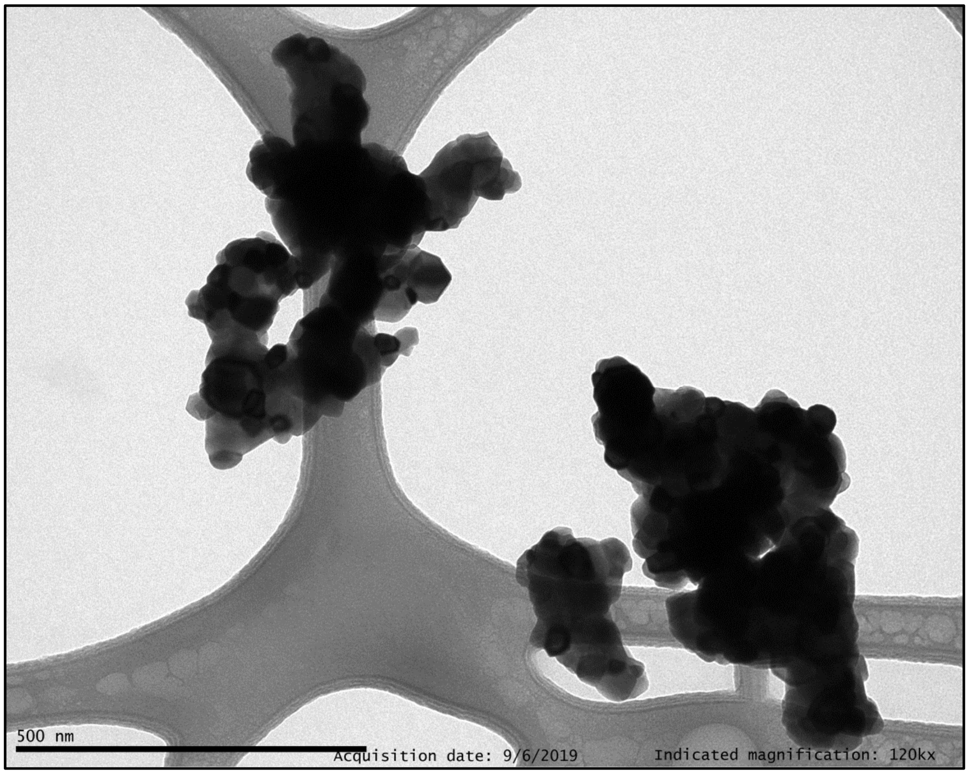

Figure S24. TEM image of Y_2O_3: Pr^{3+} NCs prepared by solvothermal method at 220 ºC for 24 h followed by calcination at 900 ºC for 4 h.

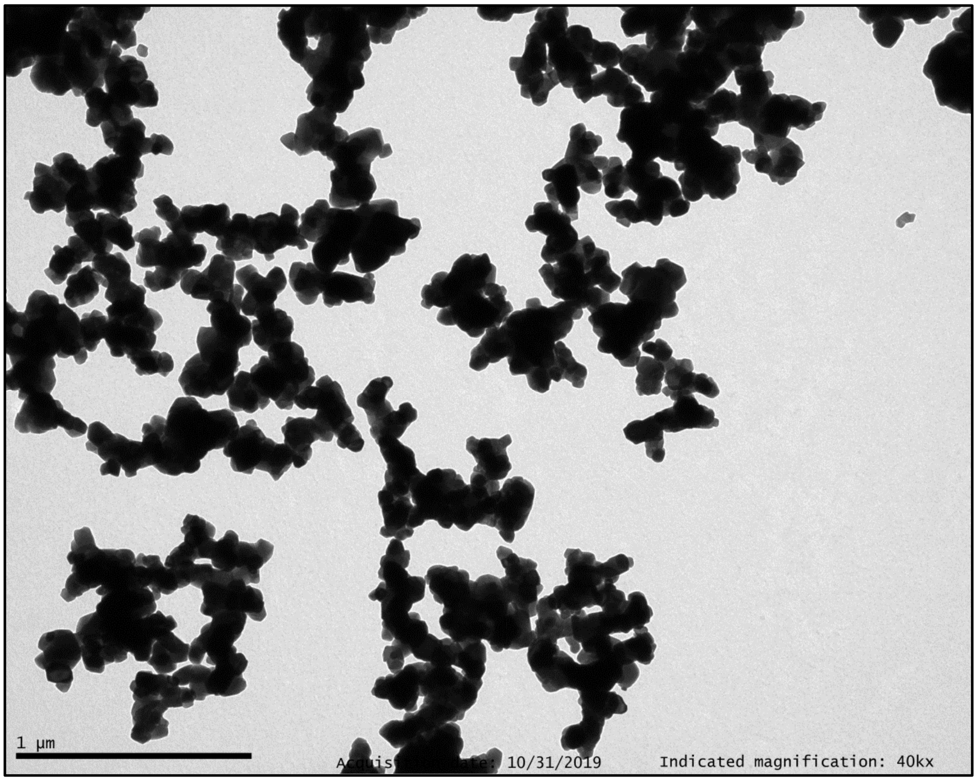

Figure S25. TEM images of Y_2O_3: Pr^{3+} NCs prepared by solvothermal method at 220 ºC for 24 h followed by calcination at 1000 ºC for 4 h.

6. Fluorescence Emission Spectroscopy

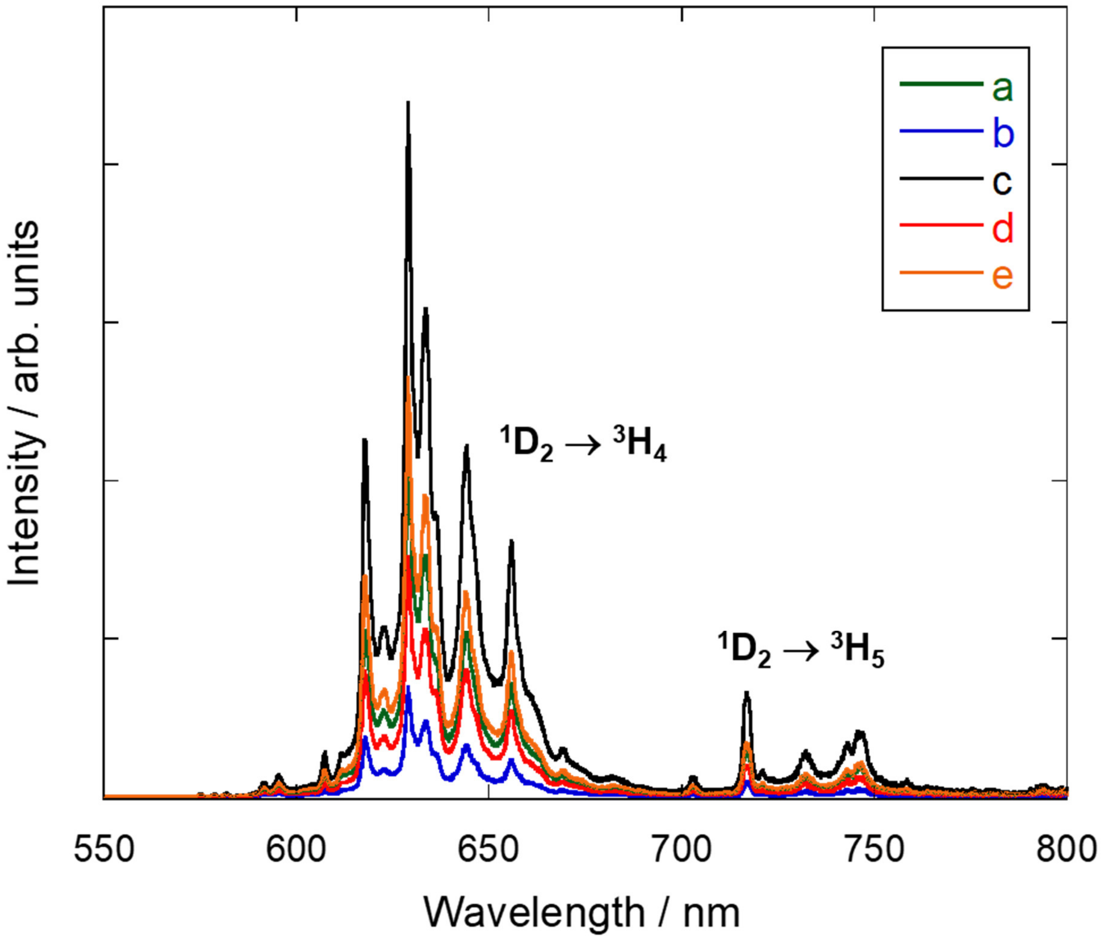

Figure S26. Emission spectra from C_2 site (λ_{ex}= 292 nm) of Y_2O_3: Pr^{3+} NCs prepared by (**a**) combustion method after calcination at 900 ºC for 4h; (**b**) molten salt method at 500 ºC followed by 10 washing cycles; (**c**) sol-gel Pechini method using citric acid as chelating agent and followed by calcination at 900 ºC for 16 h; (**d**) homogeneous precipitation using 0.832 mol of urea and 200 ml of water during 2 h reaction followed by thermal treatment at 800 ºC for 3 h; and (**e**) solvothermal method at 220 ºC followed by calcination at 1000 ºC for 4 h.

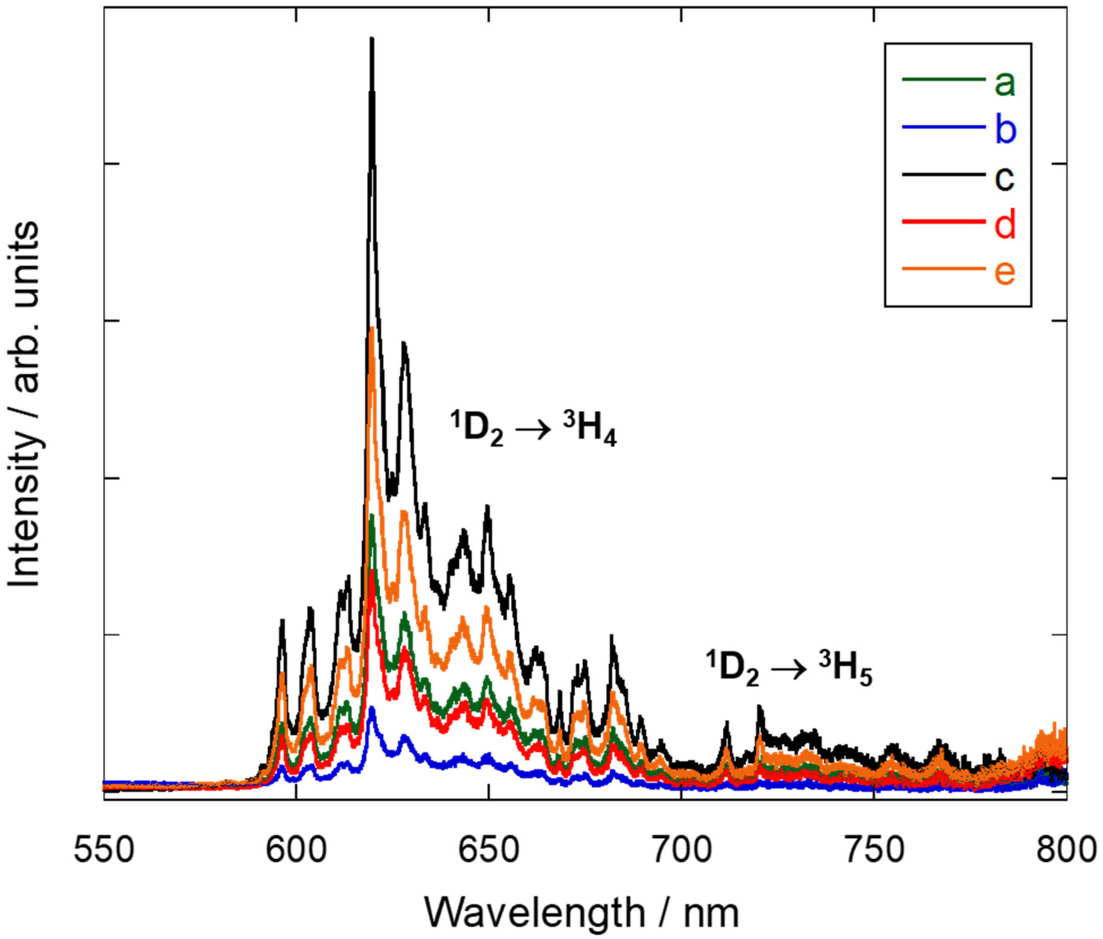

Figure S27. Emission spectra from S_6 site (λ_{ex}= 330 nm) of Y_2O_3: Pr^{3+} NCs prepared by (**a**) combustion method after calcination at 900 ºC for 4h; (**b**) molten salt method at 500 ºC followed by 10 washing cycles; (**c**) sol-gel Pechini method using citric acid as chelating agent and followed by calcination at 900 ºC for 16 h; (**d**) homogeneous precipitation using 0.832 mol of urea and 200 ml of water during 2 h reaction followed by thermal treatment at 800 ºC for 3 h; and (**e**) solvothermal method at 220 ºC followed by calcination at 1000 ºC for 4 h.

7. Fluorescence Excitation Spectroscopy

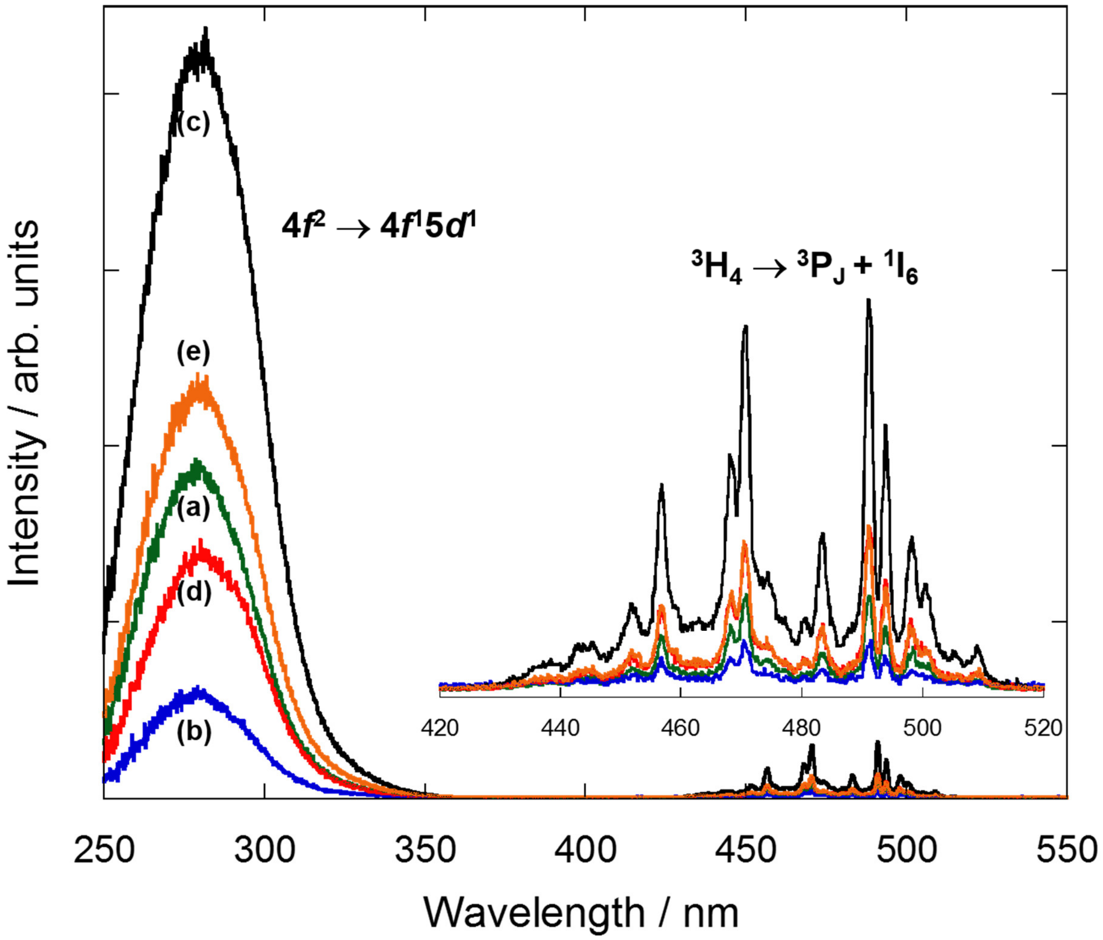

Figure S28. Excitation spectra at C_2 site (λ_{em}= 717 nm) of Y_2O_3: Pr^{3+} NCs prepared by (**a**) combustion method after calcination at 900 ºC for 4h; (**b**) molten salt method at 500 ºC followed by 10 washing cycles; (**c**) sol-gel Pechini method using citric acid as chelating agent and followed by calcination at 900 ºC for 16 h; (**d**) homogeneous precipitation using 0.832 mol of urea and 200 ml of water during 2 h reaction followed by thermal treatment at 800 ºC for 3 h; and (**e**) solvothermal method at 220 ºC followed by calcination at 1000 ºC for 4 h. The insets show a zoom of the intraconfigurational excitation transitions of Pr^{3+} ions.

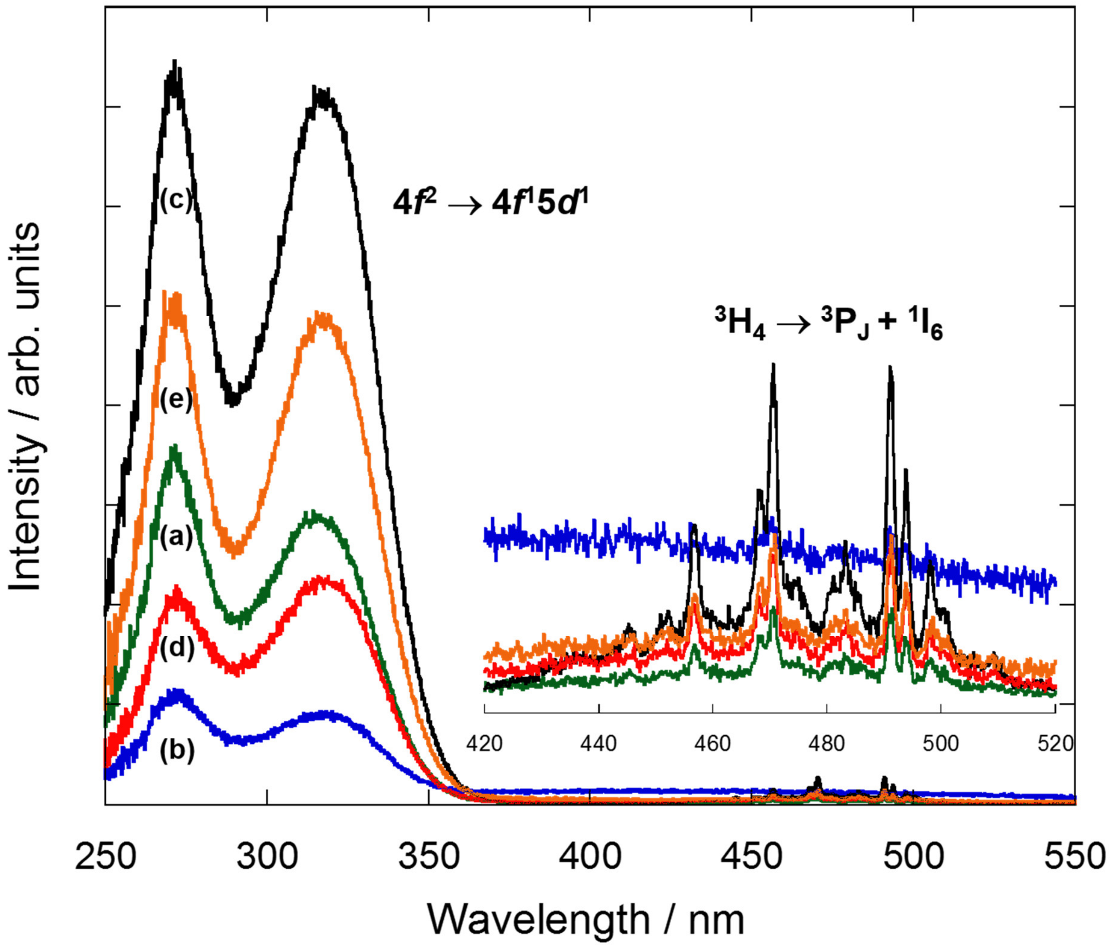

Figure S29. Excitation spectra at S_6 site (λ_{em}= 603 nm) of Y_2O_3: Pr^{3+} NCs prepared by (**a**) combustion method after calcination at 900 ºC for 4h; (**b**) molten salt method at 500 ºC followed by 10 washing cycles; (**c**) sol-gel Pechini method using citric acid as chelating agent and followed by calcination at 900 ºC for 16 h; (**d**) homogeneous precipitation using 0.832 mol of urea and 200 ml of water during 2 h reaction followed by thermal treatment at 800 ºC for 3 h; and (**e**) solvothermal method at 220 ºC followed by calcination at 1000 ºC for 4 h. The insets show a zoom of the intraconfigurational excitation transitions of Pr^{3+} ions.

8. Luminescence Lifetime (τ)

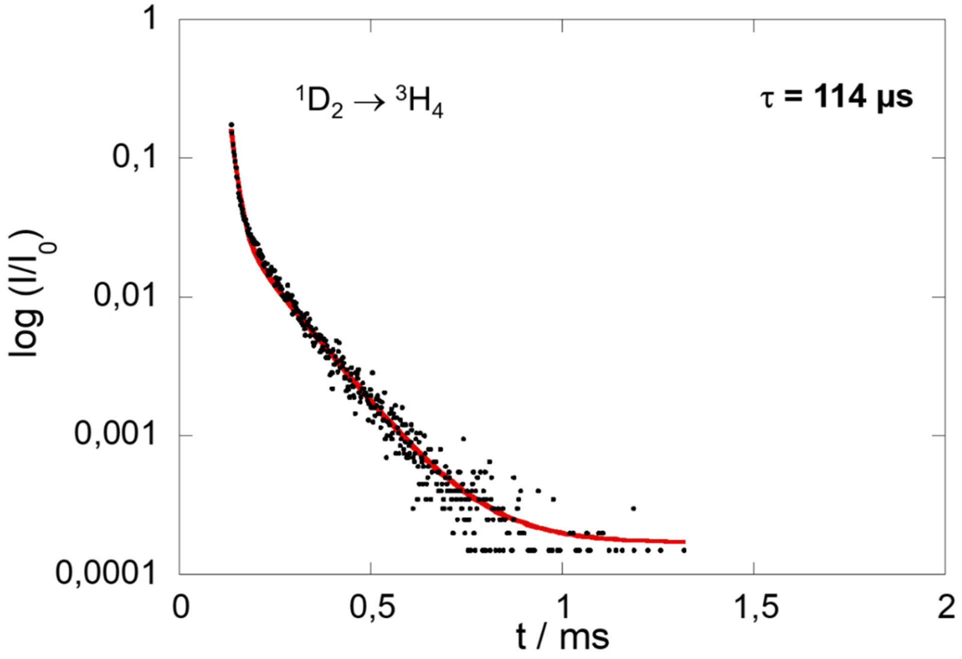

Figure S30. Luminescence decay curve of the Pr^{3+} $^1D_2 \rightarrow {}^3H_4$ emission of Y_2O_3: Pr^{3+} NCs prepared by combustion method before calcination at 900 ºC.

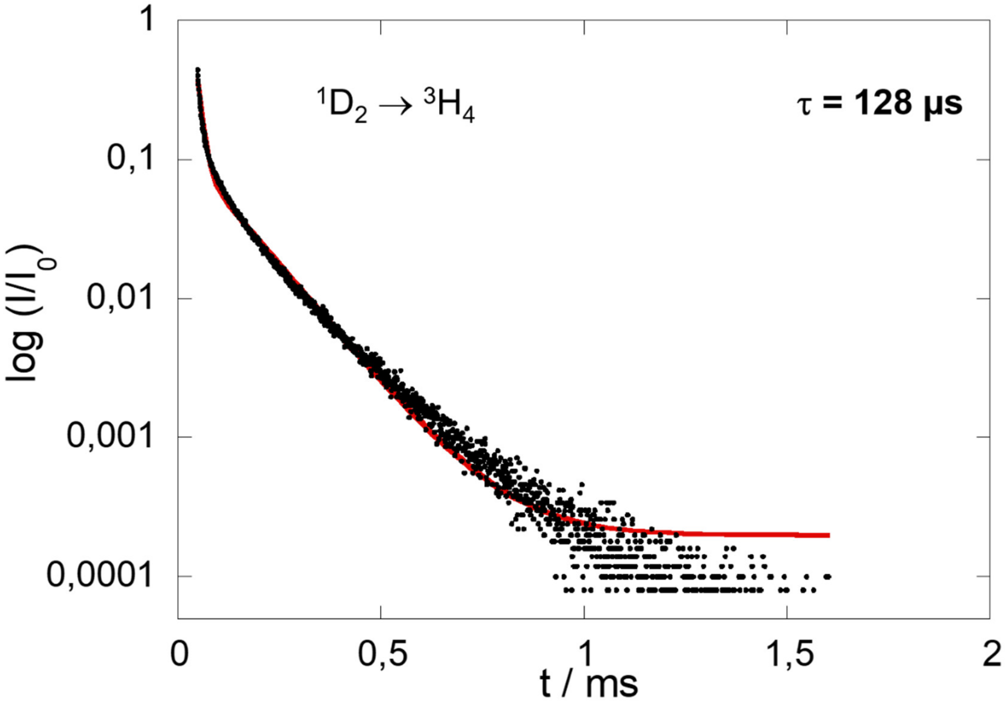

Figure S31. Luminescence decay curve of the Pr^{3+} $^1D_2 \rightarrow {}^3H_4$ emission of Y_2O_3: Pr^{3+} NCs prepared by molten salt method at 500 ºC followed by 5 washing cycles.

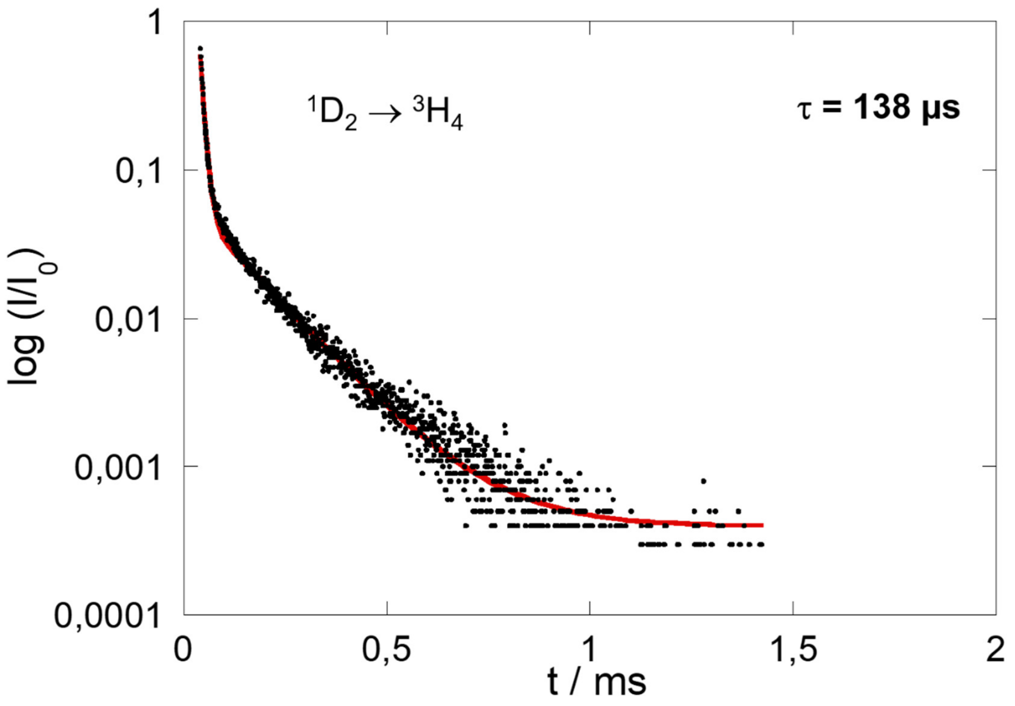

Figure S32. Luminescence decay curve of the Pr^{3+} $^1D_2 \rightarrow {}^3H_4$ emission of Y_2O_3: Pr^{3+} NCs prepared by molten salt method at 500 ºC followed by 7 washing cycles.

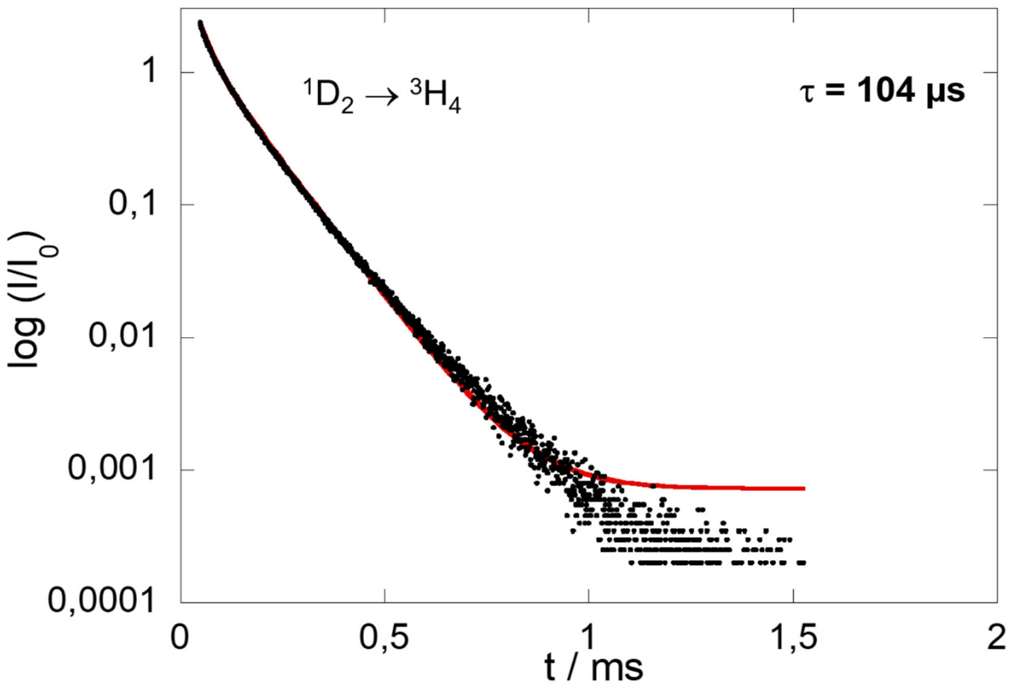

Figure S33. Luminescence decay curve of Pr^{3+} $^1D_2 \rightarrow {}^3H_4$ emission of Y_2O_3: Pr^{3+} NCs prepared by sol-gel Pechini method using citric acid as chelating agent and followed by calcination at 800 ºC for 16 h.

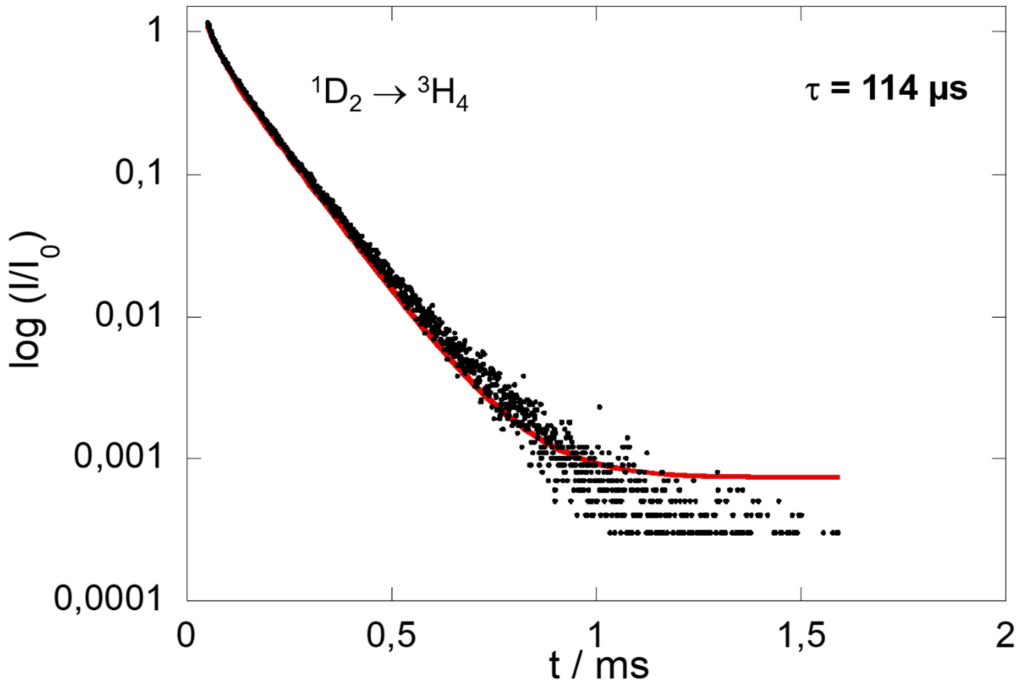

Figure S34. Luminescence decay curve of Pr^{3+} $^1D_2\rightarrow{}^3H_4$ emission of Y_2O_3: Pr^{3+} NCs prepared by sol-gel Pechini method using citric acid as chelating agent and followed by calcination at 800 ºC for 24 h.

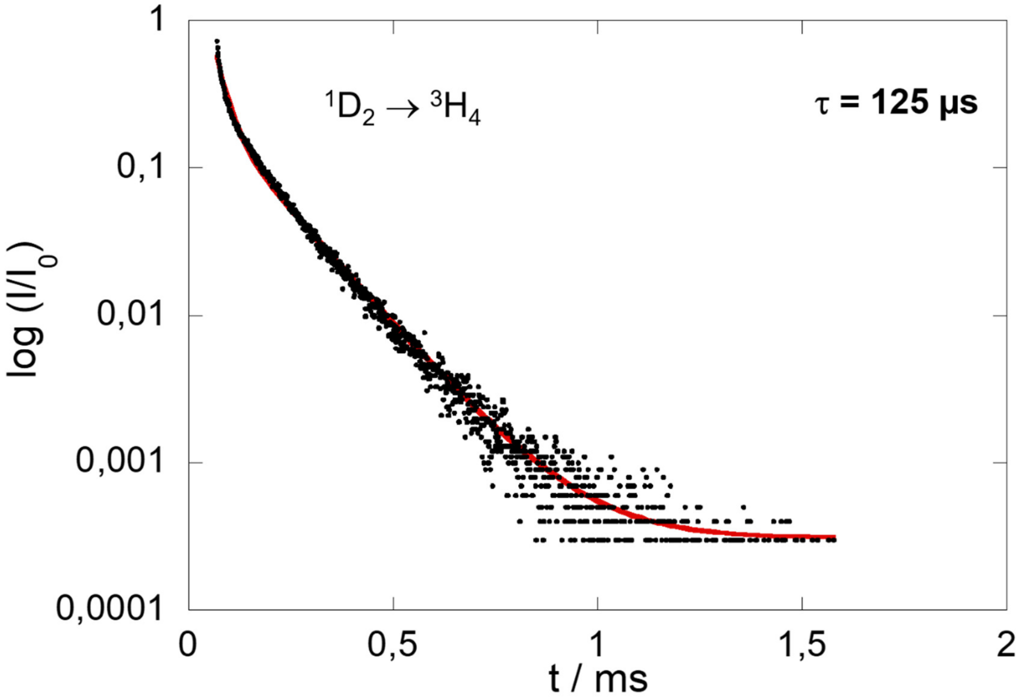

Figure S35. Luminescence decay curve of Pr^{3+} $^1D_2\rightarrow{}^3H_4$ emission of Y_2O_3: Pr^{3+} NCs prepared by sol-gel Pechini method using EDTA as chelating agent and followed by calcination at 900 ºC for 16 h.

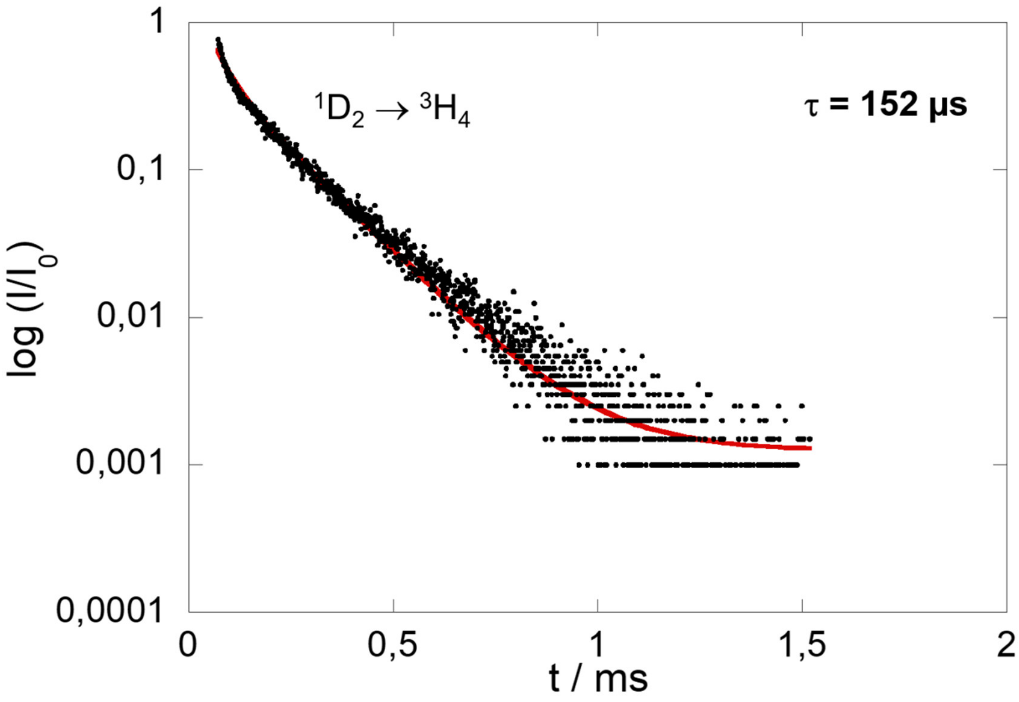

Figure S36. Luminescence decay curve of the Pr^{3+} $^1D_2\rightarrow{}^3H_4$ emission of Y_2O_3: Pr^{3+} NCs prepared by homogeneous precipitation method using 0.485 mol of urea and 360 ml of water during 2 h reaction followed by thermal treatment at 800 ºC for 3 h.

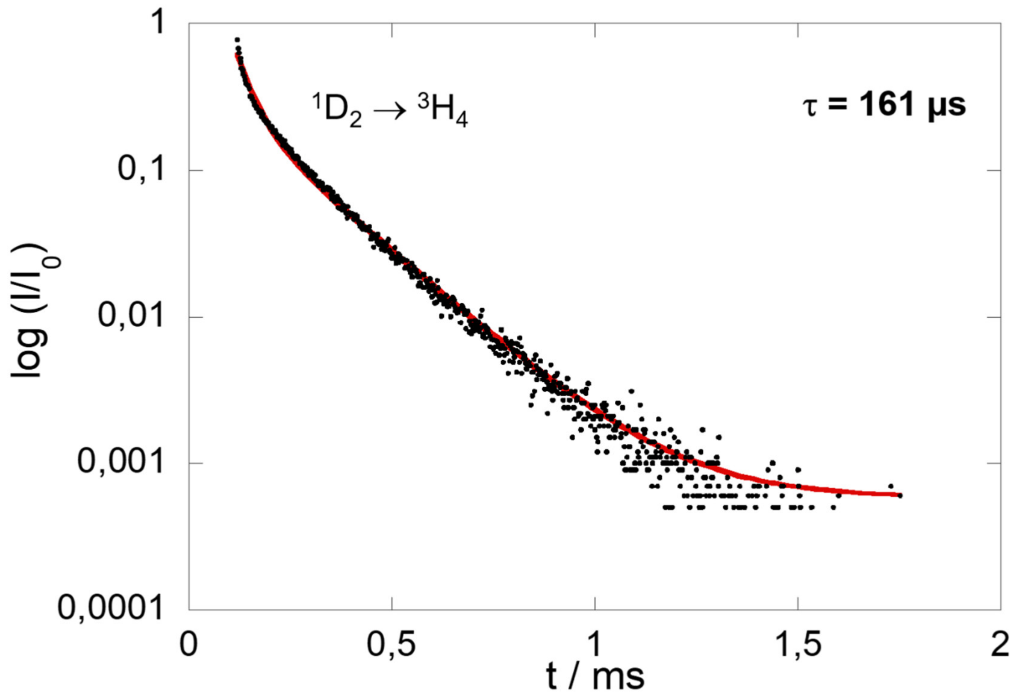

Figure S37. Luminescence decay curve of the Pr^{3+} $^1D_2\rightarrow{}^3H_4$ emission of Y_2O_3: Pr^{3+} NCs prepared by homogeneous precipitation method using 0.485 mol of urea and 200 ml of water during 2 h reaction followed by thermal treatment at 800 ºC for 3 h.

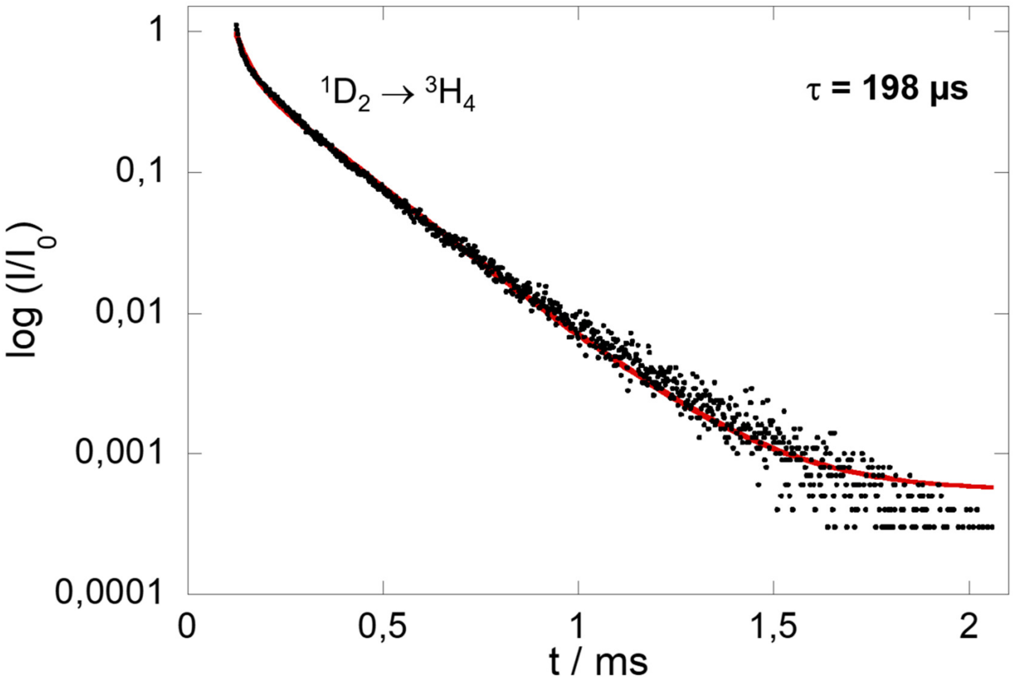

Figure S38. Luminescence decay curve of the Pr^{3+} $^1D_2 \rightarrow ^3H_4$ emission of Y_2O_3: Pr^{3+} NCs prepared by solvothermal method at 180 ºC followed by calcination at 800 ºC for 4 h.

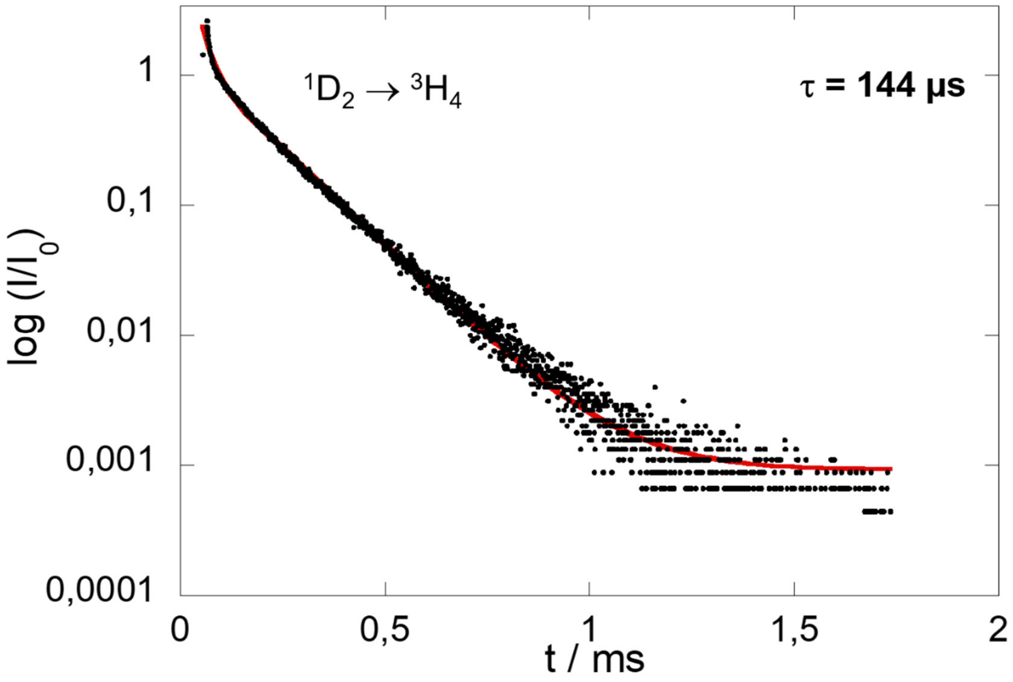

Figure S39. Luminescence decay curve of the Pr^{3+} $^1D_2 \rightarrow ^3H_4$ emission of Y_2O_3: Pr^{3+} NCs prepared by solvothermal method using 15.6957 mmol RE at 180 ºC followed by calcination at 900 ºC for 4 h.

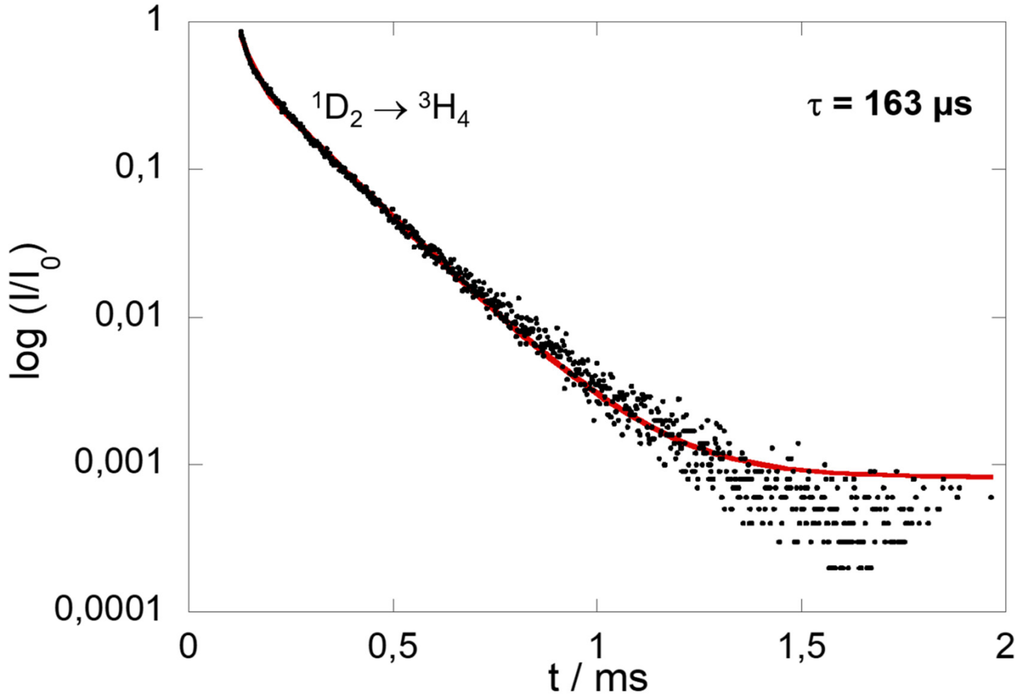

Figure S40. Luminescence decay curve of the Pr^{3+} $^1D_2{\rightarrow}^3H_4$ emission of Y_2O_3: Pr^{3+} NCs prepared by solvothermal method using EtOH at 180 ºC followed by calcination at 900 ºC for 4 h.

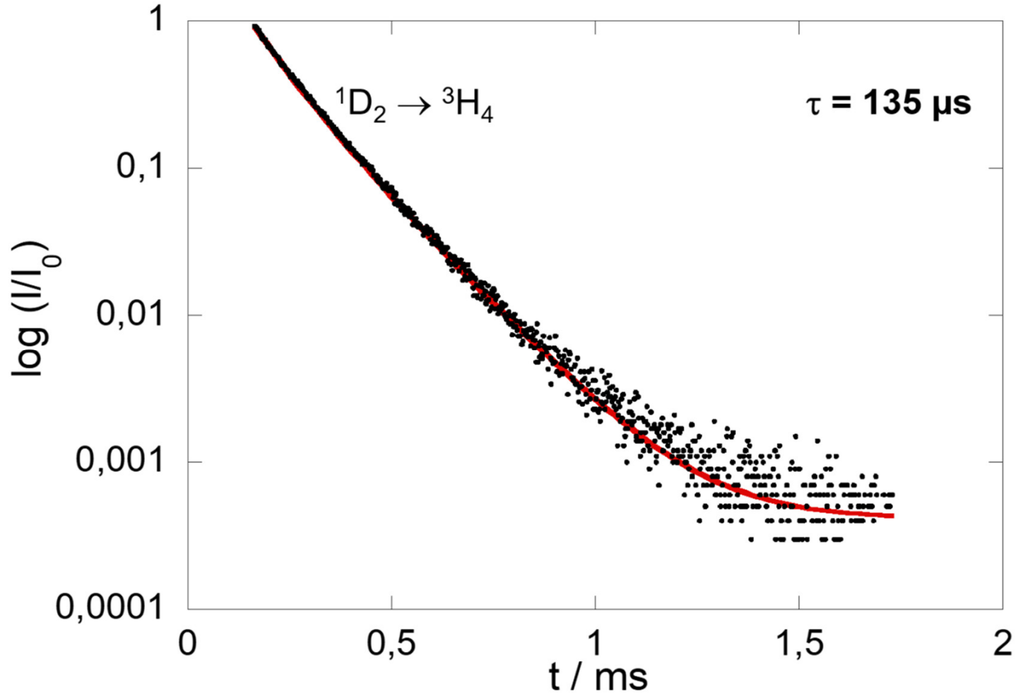

Figure S41. Luminescence decay curve of the Pr^{3+} $^1D_2{\rightarrow}^3H_4$ emission of Y_2O_3: Pr^{3+} NCs prepared by solvothermal method at 180 ºC followed by calcination at 900 ºC for 8 h.

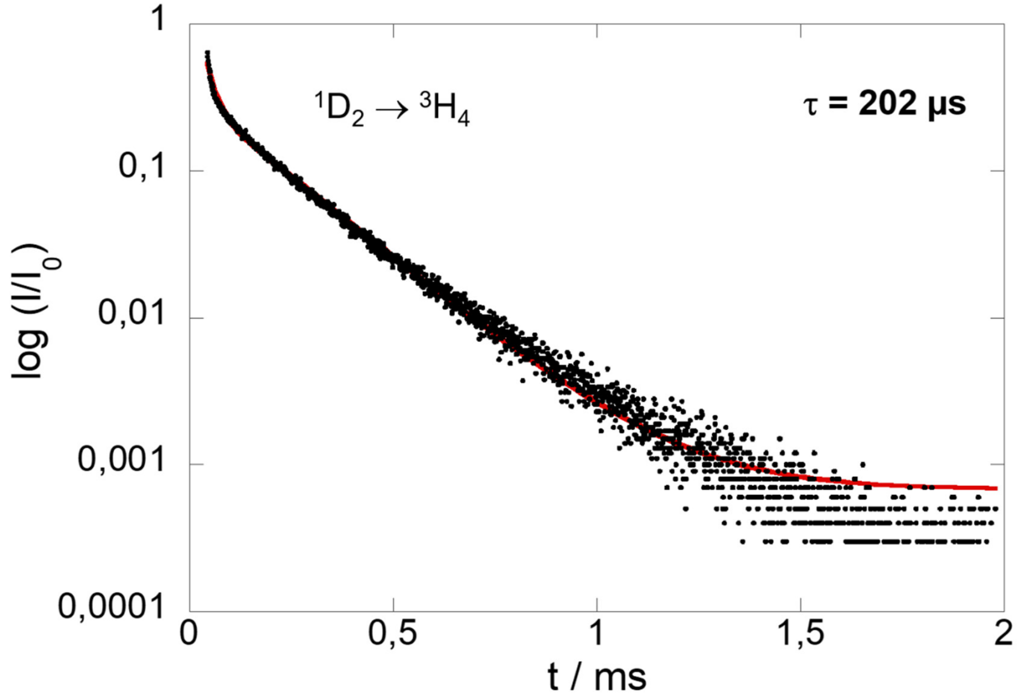

Figure S42. Luminescence decay curve of the Pr^{3+} $^1D_2 \rightarrow {}^3H_4$ emission of Y_2O_3: Pr^{3+} NCs prepared by solvothermal method at 180 ºC followed by calcination at 900 ºC for 4 h.
